# Systematic Review of the Performance of Rapid Rifampicin Resistance Testing for Drug-Resistant Tuberculosis

**DOI:** 10.1371/journal.pone.0076533

**Published:** 2013-10-03

**Authors:** Matthew Arentz, Bess Sorensen, David J. Horne, Judd L. Walson

**Affiliations:** 1 Division of Pulmonary and Critical Care Medicine, University of Washington, Seattle, Washington, United States of America; 2 Center for AIDS Research, University of Washington, Seattle, Washington, United States of America; 3 Departments of Global Health, Medicine, Pediatrics and Epidemiology, University of Washington, Seattle, Washington, United States of America; 4 Center for Clinical Research, Kenya Medical Research Institute, Nairobi, Kenya; University of Cape Town, South Africa

## Abstract

**Introduction:**

Rapid tests for rifampicin resistance may be useful for identifying isolates at high risk of drug resistance, including multidrug-resistant TB (MDR-TB). However, choice of diagnostic test and prevalence of rifampicin resistance may both impact a diagnostic strategy for identifying drug resistant-TB. We performed a systematic review to evaluate the performance of WHO-endorsed rapid tests for rifampicin resistance detection.

**Methods:**

We searched MEDLINE, Embase and the Cochrane Library through January 1, 2012. For each rapid test, we determined pooled sensitivity and specificity estimates using a hierarchical random effects model. Predictive values of the tests were determined at different prevalence rates of rifampicin resistance and MDR-TB.

**Results:**

We identified 60 publications involving six different tests (INNO-LiPA Rif. TB assay, Genotype MTBDR assay, Genotype MTBDRplus assay, Colorimetric Redox Indicator (CRI) assay, Nitrate Reductase Assay (NRA) and MODS tests): for all tests, negative predictive values were high when rifampicin resistance prevalence was ≤ 30%. However, positive predictive values were considerably reduced for the INNO-LiPA Rif. TB assay, the MTBDRplus assay and MODS when rifampicin resistance prevalence was < 5%.

**Limitations:**

In many studies, it was unclear whether patient selection or index test performance could have introduced bias. In addition, we were unable to evaluate critical concentration thresholds for the colorimetric tests.

**Discussion:**

Rapid tests for rifampicin resistance alone cannot accurately predict rifampicin resistance or MDR-TB in areas with a low prevalence of rifampicin resistance. However, in areas with a high prevalence of rifampicin resistance and MDR-TB, these tests may be a valuable component of an MDR-TB management strategy.

## Introduction

*Mycobacterium tuberculosis* (TB) remains a major causes of death and disability globally [1]. Multidrug-resistant TB (MDR-TB - defined as resistance to at least isoniazid and rifampicin), threatens to undermine TB control efforts [2]. Management of MDR-TB requires effective case detection, treatment, prevention, surveillance and monitoring [3]. However, the diagnosis of MDR-TB has remained a challenge in most countries, and less than 5% of all TB patients were tested for MDR-TB in 2010 [1].

Expanding capacity to diagnose drug-resistant TB and decreasing time to diagnosis remain priorities for global TB control. Rapid tests for rifampicin resistance may quickly identify TB patients who are likely to have MDR-TB, and the use of these tests has been recommended in high risk MDR-TB settings [4]. However, there may be significant variability in the performance of different diagnostics [5]. In addition, differences in the population prevalence of rifampicin resistance and MDR-TB may have a considerable impact on the predictive value of a strategy for rapid diagnosis of rifampicin resistance and MDR-TB.

A prior systematic review evaluated 151 published studies to determine the predictive value of rapid rifampicin resistance testing for MDR-TB [6]. This review searched only one electronic database (Medline) through 6 September 2008 for articles in English and did not use the hierarchical methods for meta-analysis recommended by the Cochrane Diagnostic Test Accuracy Working Group [7]. Several other systematic reviews have also evaluated non-commercial phenotypic and genotypic methods for rifampicin resistance but these reviews have not generated predictive values for rifampicin resistance testing as a surrogate for MDR-TB [5,8-13].

We performed a systematic review and meta-analysis to estimate the diagnostic accuracy (sensitivity and specificity) of WHO-endorsed rapid phenotypic and genotypic tests for rifampicin resistance. We reasoned that if rapid tests for rifampicin resistance detection alone could accurately predict rifampicin resistance and the conditional probability of MDR-TB is high, the use of such tests would provide a simple, and effective method for MDR-TB case identification.

## Methods

We followed guidelines for systematic reviews of diagnostic test accuracy recommended by the Cochrane Collaboration Working Group [7] and for the preferred reporting items for systematic reviews and meta-analysis (PRISMA) [14]. Because this was a systematic review of published data, no approval through the University of Washington Institutional Review Board was necessary.

### Types of studies

We included studies in populations that compared the results of the index test for rifampicin resistance with the reference standard (see definitions below). We included cross-sectional studies and cohort studies of diagnostic accuracy. We excluded studies that employed a case-control design and studies that were reported in correspondence and conference abstracts.

### Participants

We included adults and children suspected or confirmed as having TB disease, from all settings and countries. We included all direct specimens and culture isolates from humans suspected or confirmed as having M. TB.

### Index tests

We included studies that evaluated the following index tests for rifampicin resistance

#### I. Genotypic tests

• INNO-LiPA Rif. TB (Innogenetics, Ghent, Belgium)
• GenoType® MTBDR assay (Hain LifeScience GmbH, Nehren, Germany)
• GenoType® MTBDRplus assay (Hain LifeScience GmbH, Nehren, Germany)

#### II. Phenotypic tests

• Microscopic Observation Drug Susceptibility (MODS) assay
• Nitrate reductase assay (NRA)
• Colorimetric redox indicator (CRI) methods (including alamar blue, resazurin, and tetrazolium bromide)

We excluded studies that performed direct evaluation of sputum samples by the CRI methods, as the WHO does not recommend this strategy [15]. We excluded the Xpert MTB/RIF test (Cepheid, Sunnyvale, CA, USA), as a separate systematic review of this test was performed in parallel to our review [16].

### Reference Standards

We included studies that used one of the following reference standards for rifampicin resistance testing

#### I. Solid Culture

• Löwenstein-Jensen agar using the proportion, absolute concentration, or resistance ratio method
• Middlebrook 7H10 or 7H11 agar medium using the proportion, absolute concentration, or resistance ratio method

#### II. Liquid Culture

• Bactec 460 (Becton Dickinson, USA)

### Outcomes

Studies that reported data from which we could extract true positives (TP), true negatives (TN), false positives (FP), and false negatives (FN) were included. Sensitivity is the percentage of rifampicin resistant results correctly identified as rifampicin resistant when compared with the reference standard. Specificity is the percentage of rifampicin susceptible results correctly identified as rifampicin susceptible when compared with the reference standard. Positive predictive value (PPV) is the proportion of positive results that are true positives at a given prevalence of rifampicin resistance; negative predictive value (NPV) is the proportion of negative results that are true negatives at a given prevalence of rifampicin resistance.

### Search methods

We searched Medline, Embase and the Cochrane Library on 1 January 2012, using the search strategy described in the supplemental materials. We attempted to identify all studies published in English, French, or Spanish. If a prior index test systematic review had been published, we updated the systematic reviews to include studies published since prior reviews were completed. Appendix S1 in file S2 shows a representative search strategy.

### Selection of studies

Two researchers independently screened the titles and abstracts identified by electronic literature searching. Any citation identified by either of the review authors during this screen (screen 1) was selected for full-text review. Full-text papers (screen 2) were then reviewed for study eligibility using predefined inclusion and exclusion criteria. In screen 2, any discrepancies were resolved by discussion between the review authors or if they were unable to resolve, by decision of a third review author (DH). A list of excluded studies and their reasons for exclusion was maintained.

### Data extraction and management

Two researchers extracted data from each study using a piloted data extraction form. Based on the pilot, the extraction form was finalized. Two review authors then independently extracted data on the following characteristics: author, year of study, study design, patient/specimen selection, reference standard, index test, country of testing, method (indirect or direct), and the number of TP, TN, FP, and FN.

### Assessment of methodological quality

Two researchers independently assessed study quality with the Quality Assessment of Diagnostic Accuracy Studies (QUADAS) version 2 [17]. As recommended, items for each domain in the QUADAS-2 list were scored as low, high, or unclear concern of risk of bias or applicability. Appendix S3 in file S2 describes the criteria that needed to be met for each study to be rated as low, high, or unclear for each of the QUADAS-2 items.

### Statistical analysis and data synthesis

Data were entered into excel (2007, Microsoft Co, Redmond, WA). Analyses were performed using STATA/IC (version 11.0) [18]. Test sensitivity and specificity along with the 95% CI were calculated using exact methods and forest plots were generated using RevMan (version 5.1) [19]. We determined pooled sensitivity and specificity estimates using hierarchical random effects models [20] and performed these meta-analyses with the user-written command metandi in STATA/IC (version 11.0). In cases where fewer than four studies were identified, sensitivity and specificity estimates were recorded for individual studies but a meta-analysis was not performed. Predictive values were estimated under three hypothetical scenarios of rifampicin resistance prevalence (3%, 15%, and 30%) and determined mathematically as described in Table 1. Separate analyses were performed for culture isolates and clinical specimens. We also generated continuous estimates of index test predictive value across a continuum of rifampicin resistance values [21].

**Table 1:** Example of calculations for determining number of index test results classified as True Positive (TP), True Negative (TN), False Positive (FP) and False Negative (FN) per 1,000 population based on population prevalence of rifampicin resistance of 15%.

		**Reference Standard**	
		Resistance Present	Resistance Absent
Index Test	Resistance Present	TP=sensitivity X 150	FP= (1-specificity) X 850
Index Test	Resistance Absent	FN= (1-sensitivity) X 150	TN= Specificity X 850
Prevalence of Resistance: 15%		150	850

### Assessment of reporting bias

Data included in this review did not allow for formal assessment of publication bias using methods such as funnel plots or regression tests because such techniques have not been found to be helpful for diagnostic studies [7,22].

### GRADE

The quality of evidence was assessed using the GRADE approach [23]. The GRADE approach defines the quality of a body of evidence as the extent to which one can be confident that an estimate of effect or association is close to the quantity of specific interest. Quality of a body of evidence involves consideration of within-study risk of bias (methodological quality), directness of evidence, heterogeneity, precision of effect estimates and risk of publication bias. The quality rating across studies has four levels: high, moderate, low or very low. The GRADE Profiler software (version 3.6) was used for performing the GRADE analyses [24].

## Results

### Search results

**Figure 1:**
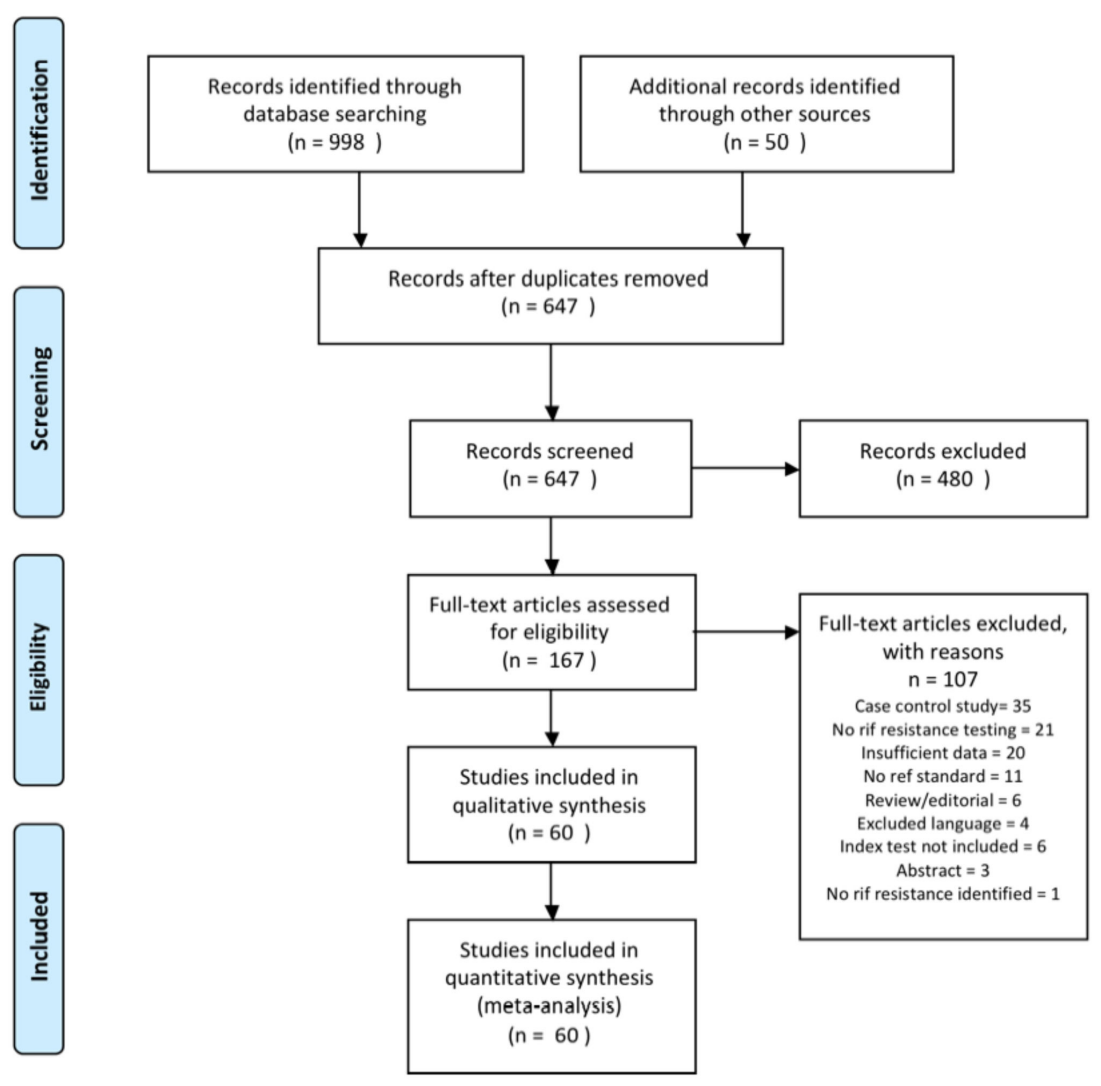
Flow of studies. Of 647 citations identified, 480 were excluded after reviewing titles and abstracts. Full-text review of the remaining 167 citations yielded 60 papers meeting eligibility criteria. Because some papers evaluated more than one rapid test, there were 62 unique studies.

Our electronic search resulted in 597 unique citations. Fifty additional citations were identified from reviewing bibliographies of previous systematic reviews [5,8-13]. Finally, 167 articles were retrieved for full-text review and 60 papers were determined to meet eligibility criteria and included in the systematic review. Because some papers included more than one index test, there were 62 datasets (hereafter referred to as studies) involving 9821 participants (Figure 1).

### Study characteristics

Studies were conducted in a variety of settings and with diverse populations. Of the total 62 studies, 19 (31%) studies evaluated genotypic tests: INNO-LiPA Rif. TB, 4 studies (6%); GenoType® MTBDR assay, 3 studies (5%); and the GenoType® MTBDRplus assay, 12 studies (19%). The remaining 43 (69%) studies evaluated the phenotypic tests: MODS, 10 studies (16%); NRA, 19 studies (31%); and CRI methods, 14 studies (23%). Characteristics of included studies are shown in appendix S2 in file S2. Appendix S4 in file S2 shows the references for included and excluded studies and reasons for exclusion. A summary of details for each index test is shown in table 2.

**Table 2:** Summary of characteristics of included studies.

**Category**	**Studies**	**No. Samples**
	**(n=62)**	**(n=9821)**
**Index test**		
INNO-LiPA Rif. TB	4	947
MTBDR	3	224
MTBDRplus	12	3337
MODS	10	1395
NRA	19	2289
CRI	14	1629
**Type of index test**		
Direct	30	5771
Indirect	32	4050
**Reference standard**		
Solid Culture	45	6995
Liquid Culture	13	2545
Both	4	281
**World Bank Country Designation for Location of Sample Collection**		
Low/Middle income	48	7705
High income	12	1738
Both	2	378
**Smear status for Studies using Clinical Specimen**		
No. smear positive	30	5118

### Methodological quality of included studies

Studies were assessed with QUADAS 2, all studies had a perfect score for flow and timing. For the domain concerning patient selection, approximately 8% of studies were considered to be at high risk of bias and 42% of studies at unknown risk of bias because these studies lacked consecutive or random selection of patients/samples, used a case-control study design, or did not report this information. For the domain concerning the index test, only 27% of studies were considered to be at low risk of bias because the study reported blinding of the index test result and the reference standard result. The domain ‘applicability of patient selection’ addresses concerns that the included patients/samples and setting do not match the review question. Of the included studies, 90% enrolled patients suspected of having TB or drug resistant TB and had low concern of bias in the applicability domain (Figure S1 in File S1).

### Genotypic tests for rifampicin resistance

Four studies, including 947 participants, were included in the analysis of the INNO-LiPA Rif. TB assay. Pooled sensitivity for rifampicin resistance was 94.1% (95% CI 86.5, 97.6) and pooled specificity was 98.8% (95% CI 93.8, 99.8) (table 3). Twelve studies, including 3337 participants, were included in the analysis of the MTBDRplus assay. Figure S2, figure S3 and figure S4 in File S1 show forest plots of sensitivity and specificity for included studies. Pooled sensitivity and pooled specificity were similar: 95.9% (95% CI 94.5, 97.0) and 98.0% (95% CI 95.1, 99.2), respectively. The MTBDR assay was not included in the meta-analysis because there were only 3 studies identified. Table 4 shows performance of genotypic index tests when used only on direct or indirect samples. Data on indeterminate test results were documented in the minority of studies. However, for the MTBDR assay, 1 study reported indeterminate results in 9% of tested samples. For the MTBDRplus assay, 3 studies reported indeterminate results in 5% of samples tested. No information on the number of indeterminate test results was documented for the INNO-LiPA Rif. TB assay.

**Table 3:** Pooled sensitivity and specificity estimates of selected phenotypic and genotypic tests for rifampicin resistance, indirect and direct testing combined.

**Test**	**Number of Studies (Participants)**	**Sensitivity % (95% CI)**	**Specificity % (95% CI)**
**INNO-LiPA Rif. TB**	4	94.1%	98.8%
	(947)	(86.5 97.6)	(93.8, 99.8)
**MTBDR**	3	N/A	N/A
	(224)		
**MTBDRplus**	11	95.9%	98.0%
	(3337)	(94.5, 97.0)	(95.1, 99.2)
**MODS**	10	98.1%	99.2%
	(1395)	(93.2, 99.5)	(94.7, 99.9)
**NRA**	19	97.7%	99.8%
	(2289)	(95.7, 98.8)	(98.9, 100.0)
**CRI**	14	99.0%	99.8%
	(1629)	(95.8, 99.8)	(98.8, 100.0)

CI = Confidence Interval

**Table 4:** Pooled sensitivity and specificity estimates for rifampicin resistance when using only direct or indirect specimens.

**Test**	**Direct Testing**			**Indirect Testing**		
	**Number of Studies**	**Sensitivity %**	**Specificity %**	**Number ofStudies**	**Sensitivity %**	**Specificity %**
	**(Participants)**	**(95% CI)**	**(95% CI)**	**(Participants)**	**(95% CI)**	**(95% CI)**
**MTBDRplus**	6	96.8%	96.4%	5	95.5%	98.5%
	(1941)	(94.1, 98.3)	(91.4, 98.5)	(960)	(91.6, 97.6)	(921, 99.7)
**NRA**	10	96.8%	99.9%	8	98.2%	99.5%
	(1465)	(93.1, 98.6)	(97.9, 100.0)	(883)	(95.6, 99.3)	(97.1, 99.9)
**MODS**	7	97.0%	99.2%	3	N/A	N/A
	(1098)	(89.1, 99.2)	(94.9, 99.9)			

CI = Confidence Interval

**Figure 2:**
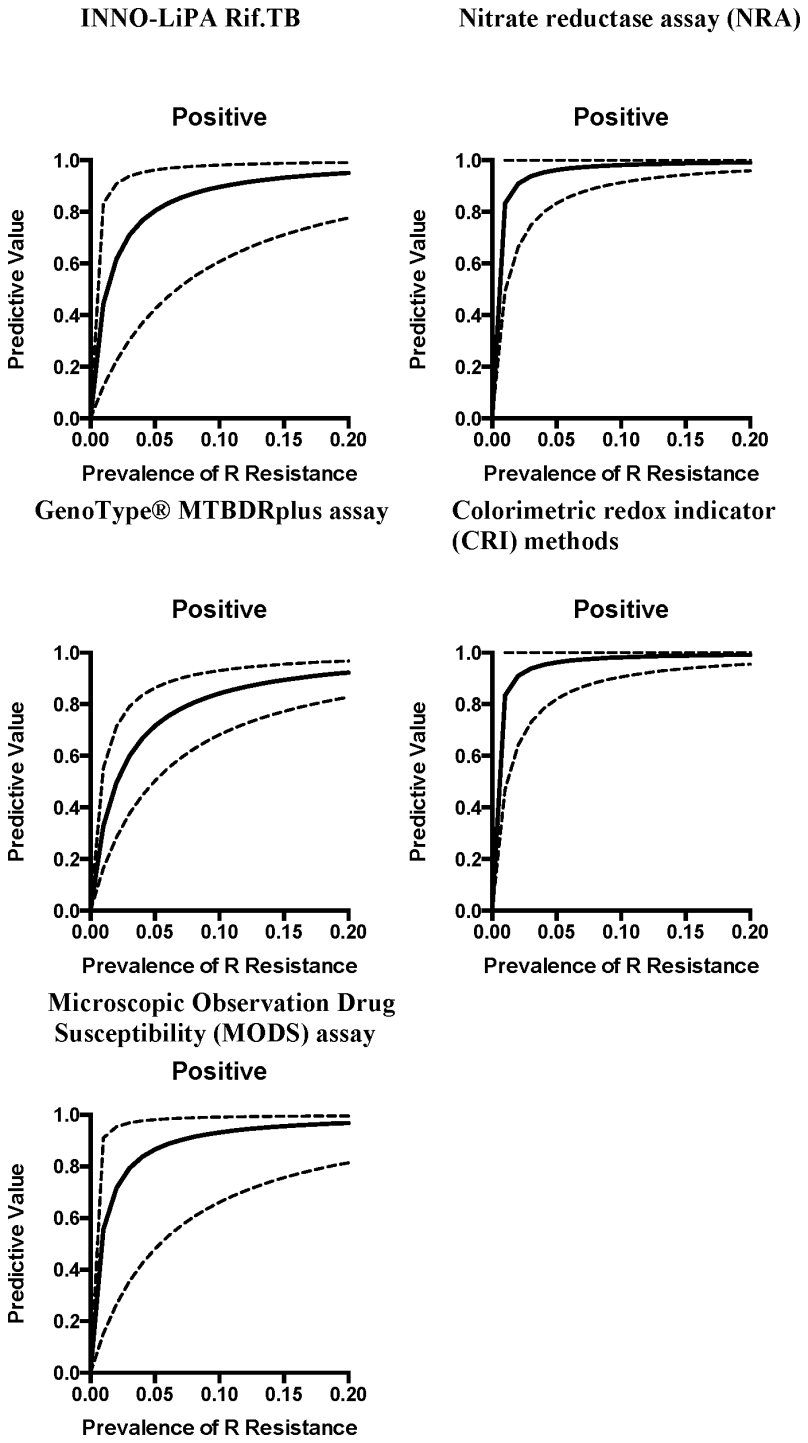
Estimates of positive predictive value, over a range of prevalence rates for rifampicin resistance (0-20% prevalence), for each index test. (dashed lines represent 95% confidence intervals around the estimate).

**Figure 3:**
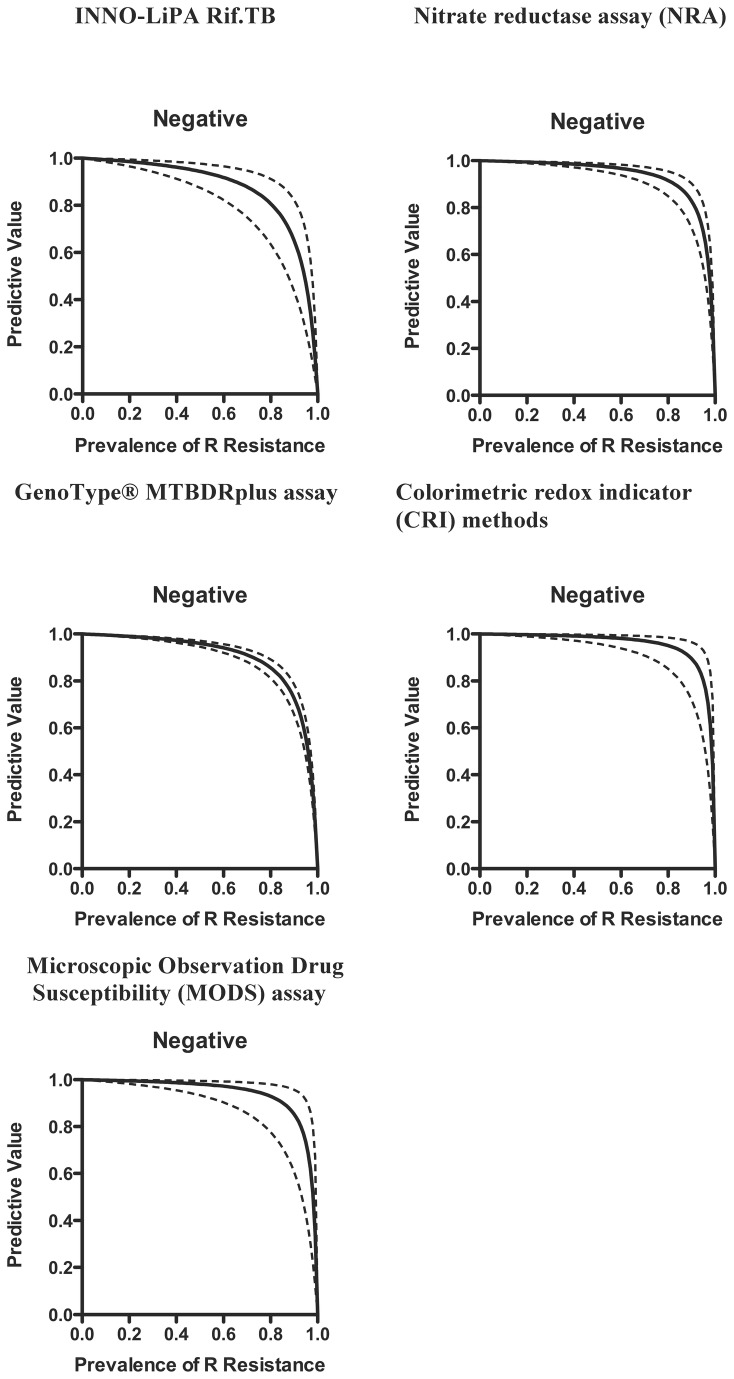
Estimates of the negative predictive value, over a range of prevalence rates for rifampicin resistance, for each index test. (dashed lines represent 95% confidence intervals around the estimate).

Each test was evaluated for predictive value assuming a rifampicin resistance prevalence of 3%, 15% and 30% in a hypothetical cohort of 1000 TB cases suspected of having MDR-TB (table 5 and table S1 in File S1). Both the INNO-LiPA Rif TB assay and the MTBDRplus assay maintained high NPVs (greater than 95%) across prevalence rates. However, at lower prevalence rates (3%), the PPV was less than 90% for both the INNO-LiPA RifTB assay (71.0%; 95% CI 31.1, 85.3) and the MTBDRplus assay (59.3%; 95% CI 37.7, 69.5). At a higher prevalence rate of 15%, the PPV was improved, 93.3%, (95% CI 72.0, 97.1) for the INNO-LiPA RifTB assay and 89.2%; 95% CI 77.5, 92.9 for the MTBDRplus assay. Figure 2 and figure 3 show positive and negative predictive values for each test along a continuum of rifampicin resistance prevalence rates in a population.

**Table 5:** Predictive values of rifampicin resistance in a hypothetic cohort of 1000 participants.

**Index Test**	**3% Rifampicin Resistance**				**15% Rifampicin Resistance**				**30% Rifampicin Resistance**			
	**PPV**	**NPV**	**FP**	**FN**	**PPV**	**NPV**	**FP**	**FN**	**PPV**	**NPV**	**FP**	**FN**
	**(95% CI)**	**(95% CI)**			**(95% CI)**	**(95% CI)**			**(95% CI)**	**(95% CI)**		
**INNO LiPA**	71.0%	99.8%	12	2	93.3%	99.0%	11	9	97.1	97.5%	9	18
	(31.1, 85.3)	(99.0, 99.9)			(72.0, 97.1)	(94.6, 99.6)			(86.2, 98.8)	(87.9, 98.9)		
**MTBDRplus**	59.3%	99.9%	21	1	89.2%	99.3%	18	6	95.3%	98.3%	15	12
	(37.7, 69.5)	(99.7, 99.9)			(77.5, 92.9)	(98.3, 99.5)			(89.3, 96.9)	(95.9, 98.9)		
**MODS**	79.8%	99.9%	8	1	95.8%	99.7%	7	3	98.2%	99.2%	5	6
	(35.5, 91.5)	(99.6, 99.9)			(75.9, 98.4)	(97.6, 99.9)			(88.4, 99.3)	(94.5 99.7)		
**NRA**	93.0%	99.9%	2	1	98.7%	99.6%	2	3	99.5%	99.0%	1	7
	(73.0, 96.7)	(99.6, 99.9)			(93.9, 99.4)	(98.0, 99.8)			(97.4, 99.8)	(95.3, 99.6)		
**CRI**	93.2%	99.9%	2	1	98.7%	99.8%	2	2	99.5%	99.5%	2	4
	(74.9, 96.8)	(99.8, 99.9)			(94.4, 99.4)	(99.2, 99.9)			(97.6, 99.8)	(98.1, 99.8)		

CI= Confidence Interval PPV=positive predictive value, NPV=negative predictive value, FP= number of false positive index test results for a cohort of 1000 patients, FN= number of false negative index test results for a cohort of 1000 patient

### Phenotypic tests for rifampicin resistance

There were 10 studies and 1395 participants included in the MODS analysis. Pooled sensitivity was 98.1% (95% CI 93.2, 99.5) and pooled specificity was 99.2% (95% CI 94.7, 99.9). The CRI assay analysis included 14 studies and 1629 participants. Sensitivity was 98.7% (95% CI 95.7, 99.6) and specificity was 99.8 (95% CI 98.9, 100.0) (table 3). Nineteen studies and 2289 participants were included in the NRA analysis, and sensitivity and specificity were calculated at 97.7% (95% CI 95.6, 98.8) and 99.8 (95% CI 99.0, 100.0), respectively. Figure S5, figure S6 and figure S7 in File S1 show forest plots of sensitivity and specificity for included studies. Table 4 shows performance of MODS and NRA when used only on direct or indirect samples. Data on indeterminate test results were documented in the minority of studies. However, for NRA, 5 studies reported indeterminate results in 6% of tested samples. No information on the number of indeterminate test results was documented for the MODS assay or the CRI assay.

Each test was then evaluated for predictive value assuming a rifampicin resistance prevalence of 3%, 15% and 30% in a hypothetical cohort of 1000 TB cases suspected of having MDR-TB (table 5 & table S1 in File S1). Similar to genotypic tests, all tests showed high NPVs (greater than 95%) across the range of prevalence rates. The PPV of the MODS assay was less than 90% at a prevalence of 3% (79.8%; 95% CI 35.5, 91.5); the PPV was greater than 90% across all prevalence rates for NRA and the CRI assay. Figure 2 and figure 3 show positive and negative predictive values for each test along a continuum of rifampicin resistance prevalence rates in a population.

## Discussion

In this systematic review and meta-analysis we evaluated the performance of WHO-endorsed rapid diagnostic tests for rifampicin resistance in comparison to conventional DST by solid or liquid culture for the detection of rifampicin resistant TB. Although we found that most of these tests perform well as a rapid marker of rifampicin resistance when prevalence is high, variability in population prevalence of rifampicin resistance and MDR-TB limited the ability of all of these tests to predict rifampicin resistance in populations with low rates of drug resistance.

These data are in agreement with estimates of sensitivity and specificity found in other systematic reviews on rapid tests for rifampicin resistance [5,6,8-11]. Because we aimed to include studies that employed ideal design for evaluation of test accuracy, we excluded case-control studies. This meant there were insufficient studies that met our inclusion criteria to evaluate performance of the MTBDR assay. In addition, the Xpert MTB/RIF test was excluded from our review, because other systematic reviews were currently reviewing performance of this test. However, other estimates of the Xpert MTB/RIF test suggest similar sensitivity and specificity, and most likely similar performance in WHO populations [16,25,26].

In light of the rapid uptake of the Xpert MTB/RIF assay in many areas, and the relatively low rates of rifampicin resistance and MDR-TB in many of the high burden TB countries, it is important to devise a strategy for improved identification of MDR-TB in low prevalence areas. In a recent review of the Xpert MTB/Rif assay, high rates of false positive rifampicin resistance test results were found in a hypothetical cohort of 1000 individuals suspected of having MDR-TB when population prevalence rates of MDR-TB were 2% [16]. One strategy for improving diagnosis of rifampicin resistance in such a cohort might be to perform a second confirmatory test for rifampicin resistance if the Xpert MTB/Rif assay indicates rifampicin resistance. Using data from a recent review, we can estimate that the Xpert MTB/Rif assay has a sensitivity of 94% and a specificity of 98% [16]. In our review, we found the MTBDRplus assay to have the lowest pooled specificity (98%). The specificity of a positive Xpert MTB/Rif assay result for rifampicin resistance AND a positive result for rifampicin resistance by MTBDRplus assay could be calculated using the following equation:

Specificity of combined positive test= 1-[(1-specificity of Xpert)x(1-specificity of MTBDRplus assay)]

Even if the prevalence of resistance was 2%, the positive predictive value of combined positive resistance testing in this setting would be 98.1% and the false positive rate would be very low (less than 2% of all positive results). Although clinical studies need to be performed to evaluate such a strategy, combined testing may be an effective way to quickly and accurately diagnose drug resistant TB.

In areas where prevalence rates of rifampicin resistance exceed 30%, these tests continued to show very good negative predictive values for rifampicin resistance and, presumably, for MDR-TB. However, we found positive predictive values to be low at prevalence rates of 15% and 3% for rifampicin resistance. This would result in the high occurrence of false positive tests at current WHO estimates of rifampicin resistance among all new TB cases. When considering test performance in a hypothetical cohort of TB cases, we found that only NRA and CRI had a positive predictive value greater than 90% when prevalence of resistance was 3%. This is in agreement with other published data evaluating the predictive value of index tests for rifampicin resistance [6].

We compared studies that evaluated only direct or indirect samples, and found similar test sensitivity and specificity for the MTBDRplus assay and NRA. We had insufficient data to perform this sub-group analysis for the INNO-LiPA Rif. TB assay or the MODS assay. In addition, although the NRA and CRI methods were observed to perform best in the included studies, GRADE evaluation of the included studies for these tests found very serious limitations in blinding and study design, which may have had a significant effect on sensitivity and specificity estimates.

If one considers how these tests would perform in identifying MDR-TB in TB populations, there are a number of important issues to account for. First, we only evaluated test performance for rifampicin resistance and presumed a strong correlation between rifampicin resistance and MDR-TB. In reality, the conditional probability that a TB case has MDR-TB when rifampicin resistance is present will vary by setting. Others have reported that this conditional probability may be low in some settings, although updated estimates are necessary [27]. In our review, the conditional probability of MDR-TB was 93.6%. Second, when considering global estimates of rifampicin resistance (6%) and MDR-TB (5.1%) our tests PPV suggest there will be a high likelihood of false positive test rests in many low prevalence settings (with false positives accounting for 16-28% of all positive test results at a prevalence of resistance at 5.1%). Among the 22 high burden TB countries which account for 80% of the world’s burden of TB, the WHO estimates only Afghanistan, China, and the Russian Federation have MDR-TB prevalence rates above this level. This suggests that these tests will perform best as a surrogate test for MDR-TB in targeted populations with higher rates of drug resistance, and studies which further identify patients at high-risk for drug resistance are needed to provide more accurate results.

Overall, the decision of how to use best use rapid tests for rifampicin resistance in an MDR-TB treatment program remains unclear. When considering a positive rapid rifampicin resistance test result in a TB patient, results from conventional drug sensitivity testing (DST) via solid or liquid culture will likely play a large role in identifying the ideal treatment regimen. While awaiting DST results in a patient with a high risk of MDR-TB or a high risk of treatment failure, use of an MDR-TB regimen with isoniazid may be appropriate if resources permit. However, further studies are needed which describe the comparative costs and outcome benefits of such a strategy. In the interim, our review adds further support to the current WHO recommendation that all patient’s who have rapid drug resistance testing also undergo full phenotypic DST.

This review has several strengths, including a broad search strategy and inclusion of papers in multiple languages. We used rigorous statistical methods in our pooled estimates of sensitivity and specificity. In addition, we limited study design to include only cross sectional studies, providing a better estimate of the diagnostic accuracy of each index test when compared to the reference standard.

This review also has several limitations. With regards to the MTBDRplus assay, many manuscripts did not specify whether version 1 or version 2 of the assay was used, and this may have affected specificity and sensitivity estimates. Also, only the minority of studies recorded information on indeterminate test results. High rates of indeterminate results could significantly affect test utility even if test performance is excellent. In many studies we were unclear whether patient selection or index test performance could have introduced bias. Ideally diagnostic tests should enroll a consecutive or random sample of eligible patients and blind the operator of the index test to reference standard results. We also did not evaluate critical concentration thresholds for the colorimetric test studies and these results should be interpreted with caution. We were unable to identify many studies that performed a direct comparison of index tests for rifampicin resistance. For this reason, the GRADE quality of the studies was low or very low in most cases, with only MDRTBplus and MODS being graded as moderate quality of evidence (table S2 and table S3 in File S1).

## Conclusions

This systematic review updates previous estimates on the performance of rapid tests for identifying rifampicin resistance. It describes limitations in test PPV for rifampicin resistance and MDR-TB in the WHO high burden TB countries. Further studies are needed to better describe and compare test performance as a predictor of rifampicin resistance and MDR-TB and describe the costs and benefits of a rapid strategy for identifying and treating drug resistance.

## Supporting Information

Checklist S1 **PRISMA checklist.**
(DOC)

File S1 Figure S1, Assessment of study quality. Figure S2, Forest plot: Sensitivity and Specificity of the INNO LiPA. Figure S3, Forest plot: Sensitivity and Specificity of the MTBDR assay. Figure S4, Forest plot: Sensitivity and Specificity of the MTBDRplus assay. Figure S5, Forest plot: Sensitivity and Specificity of the MODS assay. Figure S6, Forest plot: Sensitivity and Specificity of the Nitrate Reductase assay. Figure S7, Forest plot: Sensitivity and Specificity of the CRI assay. Table S1, Predictive values of rifampicin resistance in a hypothetic cohort of 1000 participants stratified by test method. Table S2, A. GRADE Evidence Profiles Outcome: INNO-LiPA Rif. TB as a replacement test for conventional drug susceptibility testing of rifampicin resistance. B. GRADE Evidence Profiles Outcome: MTBDR as a replacement test for conventional drug susceptibility testing of rifampicin resistance. C. GRADE Evidence Profiles Outcome: MTBDRplus as a replacement test for conventional drug susceptibility testing of rifampicin resistance. D. GRADE Evidence Profiles Outcome: MODS as a replacement test for conventional drug susceptibility testing of rifampicin resistance. E. GRADE Evidence Profiles Outcome: Nitrate reductase assay (NRA) as a replacement test for conventional drug susceptibility testing of rifampicin resistance. F. GRADE Evidence Profiles Outcome: Colorimetric redox indicator (CRI) assays as a replacement test for conventional drug susceptibility testing of rifampicin resistance. Table S3, GRADE Summary of Findings.
(DOC)

File S2 Appendix S1, Search strategy. Appendix S2, Characteristics of included studies. Appendix S3, Methodological Quality Assessment. QUADAS 2 evaluation. Appendix S4, references for included and excluded studies.
(DOC)

## References

[1] WHO (2011) Global Tuberculosis Control 2011. Geneva, Switzerland: World Health Organization.

[2] Arentz M, Walson JL (2011) Tuberculosis. In: HAaM, Juergensmeyer. The Encyclopedia of Global Studies. Thousand Oaks, CA: Sage Publications.

[3] WHO (2011) Guidelines for the progrommatic management of drug-resistant tuberculosis: 2011 update. Geneva, Switzerland: World Health Organization. 23844450

[4] WHO (2008) Policy guidance on drug-susceptibility testing (DST) of second-line antituberculosis drugs. Geneva, Switzerland: World Health Organization. 26290924

[5] Bwanga F, Hoffner S, Haile M, Joloba ML (2009) Direct susceptibility testing for multi drug resistant tuberculosis: a meta-analysis. BMC Infect Dis 9: 67. doi:10.1186/1471-2334-9-67. PubMed: 19457256. 19457256 PMC2696456

[6] Chang KC, Yew WW, Zhang Y (2009) A systematic review of rapid drug suscepbility tests for multidrug-resistant tuberculosis using rifampin resistance as a surrogate. Expert Opin Med Diagn 3: 99-122. doi:10.1517/17530050802665694. PubMed: 23485158. 23485158

[7] Macaskill P, [!(surname)!], Deeks JJ, Harbord RM, Takwoingi Y (2010) Analysing and Presenting Results10. In: Cochrane Handbook for Systematic Reviews of Diagnostic Test Accuracy Chapter Version 0.9.0 Deeks JJ BP, Gatsonis C editor: The Cochrane Collaboration

[8] Ling DI, Zwerling AA, Pai M (2008) GenoType MTBDR assays for the diagnosis of multidrug-resistant tuberculosis: a meta-analysis. Eur Respir J 32: 1165-1174. doi:10.1183/09031936.00061808. PubMed: 18614561. 18614561

[9] Martin A, Panaiotov S, Portaels F, Hoffner S, Palomino JC et al. (2008) The nitrate reductase assay for the rapid detection of isoniazid and rifampicin resistance in Mycobacterium tuberculosis: a systematic review and meta-analysis. J Antimicrob Chemother 62: 56-64. doi:10.1093/jac/dkn139. PubMed: 18407918. 18407918

[10] Martin A, Portaels F, Palomino JC (2007) Colorimetric redox-indicator methods for the rapid detection of multidrug resistance in Mycobacterium tuberculosis: a systematic review and meta-analysis. J Antimicrob Chemother 59: 175-183. PubMed: 17135182. 17135182 10.1093/jac/dkl477

[11] Minion J, Leung E, Menzies D, Pai M (2010) Microscopic-observation drug susceptibility and thin layer agar assays for the detection of drug resistant tuberculosis: a systematic review and meta-analysis. Lancet Infect Dis 10: 688-698. doi:10.1016/S1473-3099(10)70165-1. PubMed: 20813587. 20813587

[12] Morgan M, Kalantri S, Flores L, Pai M (2005) A commercial line probe assay for the rapid detection of rifampicin resistance in Mycobacterium tuberculosis: a systematic review and meta-analysis. BMC Infect Dis 5: 62. doi:10.1186/1471-2334-5-62. PubMed: 16050959. 16050959 PMC1185540

[13] Leung E, Minion J, Benedetti A, Pai M, Menzies D (2012) Microcolony culture techniques for tuberculosis diagnosis: a systematic review. Int J Tuberc Lung Dis 16: 16-23, i-iii doi:10.5588/ijtld.10.0065. PubMed: 21986554. 21986554

[14] Moher D, Liberati A, Tetzlaff J, Altman DG (2009) Preferred reporting items for systematic reviews and meta-analyses: the PRISMA statement. J Clin Epidemiol 62: 1006-1012. doi:10.1016/j.jclinepi.2009.06.005. PubMed: 19631508. 19631508

[15] WHO (2011) Noncommercial culture and drug-susceptibility testing methods for screening patients at risk for multidrug-resistant tuberculosis: policy statement. Geneva. 23586116

[16] Steingart KR, Sohn H, Schiller I, Kloda LA, Boehme CC et al. (2013) Xpert(R) MTB/RIF assay for pulmonary tuberculosis and rifampicin resistance in adults. Cochrane Database Syst Rev 1: CD009593. 10.1002/14651858.CD009593.pub2 PMC4470352 23440842

[17] Whiting PF, Rutjes AW, Westwood ME, Mallett S, Deeks JJ et al. (2011) QUADAS-2: a revised tool for the quality assessment of diagnostic accuracy studies. Ann Intern Med 155: 529-536. doi:10.7326/0003-4819-155-8-201110180-00009. PubMed: 22007046. 22007046

[18] StataCorp (2009) Stata Statistical Software, Release 11. TX: College Station.

[19] Collaboration TC (2011). Rev Manag (RevMan). 5.1 ed. Copenhagen: The Nordic Cochrane Center.

[20] Rutter CM, Gatsonis CA (2001) A hierarchical regression approach to meta-analysis of diagnostic test accuracy evaluations. Stat Med 20: 2865-2884. doi:10.1002/sim.942. PubMed: 11568945. 11568945

[21] Macaskill P, [!(surname)!], Deeks JJ, Harbord RM, Takwoingi Y (2010) Analysing and Presenting Results. In: Cochrane handbook for systematic reviews of diagnostic test accuracy. London: The Cochrane Collaboration.

[22] Tatsioni A, Zarin DA, Aronson N, Samson DJ, Flamm CR et al. (2005) Challenges in systematic reviews of diagnostic technologies. Ann Intern Med 142: 1048-1055. doi:10.7326/0003-4819-142-12_Part_2-200506211-00004. PubMed: 15968029. 15968029

[23] Guyatt GH, Oxman AD, Vist GE, Kunz R, Falck-Ytter Y et al. (2008) GRADE: an emerging consensus on rating quality of evidence and strength of recommendations. BMJ 336: 924-926. doi:10.1136/bmj.39489.470347.AD. PubMed: 18436948. 18436948 10.1136/bmj.39489.470347.AD PMC2335261

[24] Brozek J, [!(surname)!], Schünemann H (2008) GRADEpro, 3.6 for Windows McMaster University.

[25] Boehme CC, Nicol MP, Nabeta P, Michael JS, Gotuzzo E et al. (2011) Feasibility, diagnostic accuracy, and effectiveness of decentralised use of the Xpert MTB/RIF test for diagnosis of tuberculosis and multidrug resistance: a multicentre implementation study. Lancet 377: 1495-1505. doi:10.1016/S0140-6736(11)60438-8. PubMed: 21507477. 21507477 PMC3085933

[26] Lawn SD, Brooks SV, Kranzer K, Nicol MP, Whitelaw A et al. (2011) Screening for HIV-associated tuberculosis and rifampicin resistance before antiretroviral therapy using the Xpert MTB/RIF assay: a prospective study. PLOS Med 8: e1001067 PubMed: 21818180. 21818180 10.1371/journal.pmed.1001067 PMC3144215

[27] Smith SE, Kurbatova EV, Cavanaugh JS, Cegielski JP (2012) Global isoniazid resistance patterns in rifampin-resistant and rifampin-susceptible tuberculosis. Int J Tuberc Lung Dis 16: 203-205. doi:10.5588/ijtld.11.0445. PubMed: 22136739. 22136739 PMC4593497

