# Supplementary material for: Systematic Review of the Performance of Rapid Rifampicin Resistance Testing for Drug-Resistant Tuberculosis
Source: PLoS One. 2013 Oct 3;8(10):e76533. doi: 10.1371/journal.pone.0076533 (PMC3789679; doi:10.1371/journal.pone.0076533)
Supplement: File S1 — Figure S1, Assessment of study quality. Figure S2, Forest plot: Sensitivity and Specificity of the INNO LiPA. Figure S3, Forest plot: Sensitivity and Specificity of the MTBDR assay. Figure S4, Forest plot: Sensitivity and Specificity of the MTBDRplus assay. Figure S5, Forest plot: Sensitivity and Specificity of the MODS assay. Figure S6, Forest plot: Sensitivity and Specificity of the Nitrate Reductase assay. Figure S7, Forest plot: Sensitivity and Specificity of the CRI assay. Table S1, Predictive values of rifampicin resistance in a hypothetic cohort of 1000 participants stratified by test method. Table S2, A. GRADE Evidence Profiles Outcome: INNO-LiPA Rif. TB as a replacement test for conventional drug susceptibility testing of rifampicin resistance. B. GRADE Evidence Profiles Outcome: MTBDR as a replacement test for conventional drug susceptibility testing of rifampicin resistance. C. GRADE Evidence Profiles Outcome: MTBDRplus as a replacement test for conventional drug susceptibility testing of rifampicin resistance. D. GRADE Evidence Profiles Outcome: MODS as a replacement test for conventional drug susceptibility testing of rifampicin resistance. E. GRADE Evidence Profiles Outcome: Nitrate reductase assay (NRA) as a replacement test for conventional drug susceptibility testing of rifampicin resistance. F. GRADE Evidence Profiles Outcome: Colorimetric redox indicator (CRI) assays as a replacement test for conventional drug susceptibility testing of rifampicin resistance. Table S3, GRADE Summary of Findings. (DOC) [file pone.0076533.s002.doc]

**Table S1.** Predictive values of rifampicin resistance in a hypothetic cohort of 1000 participants stratified by test method

| **Test Method** | **Index Test** | **3% Rifampicin Resistance** | | | | **15% Rifampicin Resistance** | | | | **30% Rifampicin Resistance** | | | |
| --- | --- | --- | --- | --- | --- | --- | --- | --- | --- | --- | --- | --- | --- |
|  |  | **PPV**  **(95% CI)** | **NPV**  **(95% CI)** | **FP** | **FN** | **PPV**  **(95% CI)** | **NPV**  **(95% CI)** | **FP** | **FN** | **PPV**  **(95% CI)** | **NPV**  **(95% CI)** | **FP** | **FN** |
| **Direct Samples** | **MTBDRplus** | 45.4%  (25.5, 56.7) | 99.9%  (99.8, 99.9) | 36 | 1 | 82.6%  (66.2, 88.2) | 99.4 %  (98.6, 99.6) | 32 | 5 | 92.0  (82.6, 94.8) | 98.6%  (96.7, 99.1) | 26 | 10 |
|  | **NRA** | 96.0%  (58.4, 99.0) | 99.9%  (98.3, 99.9) | 1 | 1 | 99.3%  (88.9, 99.8) | 99.4%  (91.2, 99.9) | 1 | 5 | 99.7%  (95.1, 99.9) | 98.7%  (81.0, 99.7) | 1 | 10 |
|  | **MODS** | 79.6%  (36.5, 91.2) | 99.9%  (99.4, 99.9) | 8 | 1 | 95.7%  (76.6, 98.3) | 99.5%  (96.5, 99.8) | 7 | 5 | 98.2%  (88.8, 99.3) | 98.7%  (91.9, 99.5) | 5 | 9 |
| **Indirect Samples** | **MTBDRplus** | 66.9%  (26.3, 83.0) | 99.9%  (99.2, 99.9) | 15 | 1 | 92.0%  (67.1, 96.5) | 99.2%  (95.6, 99.7) | 13 | 7 | 96.6%  (83.2, 98.5) | 98.1%  (90.0, 99.2) | 11 | 14 |
|  | **NRA** | 85.2%  (50.5, 93.3) | 99.9%  (99.7, 99.9) | 5 | 1 | 97.1%  (85.3 98.8) | 99.7%  (98.3, 99.9) | 5 | 3 | 98.8%  (93.3 99.5) | 99.2%  (95.9, 99.7) | 4 | 5 |

**CI=Confidence Interval PPV=positive predictive value, NPV=negative predictive value, FP= number of false positive index test results for a cohort of 1000 patients, FN= number of false negative index test results for a cohort of 1000 patient**

**Table S2. GRADE Evidence Profiles:**

**Table S2A. Outcome: INNO-LiPA Rif.TB as a replacement test for conventional drug susceptibility testing of rifampicin resistance^A1^**

| **No of Participants (Studies)** | **Study design** | | **Limitations** | **Indirectness (applicability)** | **Inconsistency** | **Imprecision** | **Publication Bias** | **Quality of Evidence (GRADE)** | **Importance** |
| --- | --- | --- | --- | --- | --- | --- | --- | --- | --- |
| **True positives** | | | | | | | | |  |
| 100 (4) | Cross-sectional | | Serious^A2^  (-1) | Serious^A3^  (-1) | No Serious Inconsistency^A4^ | No Serious Imprecision^A5^ | Strongly Suspected^A6^ | Low ⊕⊕🌕🌕 | Critical (7-9) |
| **True negatives** | | | | | | | | |  |
| 835 (4) | Cross-sectional | | Serious^A2^  (-1) | Serious^A3^  (-1) | No Serious Inconsistency^A4^ | No Serious Imprecision^A5^ | Strongly Suspected^A6^ | Low ⊕⊕🌕🌕 | Critical (7-9) |
| **False positives** | | | | | | | | |  |
| 6 (4) | Cross-sectional | | Serious^A2^  (-1) | Serious^A3^  (-1) | No Serious Inconsistency^A4^ | No Serious Imprecision^A5^ | Strongly Suspected^A6^ | Low ⊕⊕🌕🌕 | Critical (7-9) |
| **False negatives** | | | | | | | | |  |
| 6 (4) | | Cross-sectional | Serious^A2^  (-1) | Serious^A3^  (-1) | No Serious Inconsistency^A4^ | No Serious Imprecision^A5^ | Strongly Suspected^A6^ | Low ⊕⊕🌕🌕 | Critical (7-9) |

Based on pooled sensitivity = 94% (95% CI 87, 98) and pooled specificity = 99% (95% CI 94, 100)

**Footnotes**

^A1^As recommended, we rated the quality of evidence as high (no points subtracted), moderate (1 point subtracted), low (2 points subtracted), or very low (>2 points subtracted) based on five criteria: imitations, indirectness, inconsistency, imprecision, and publication bias. For each outcome, the quality of evidence started at high when there were randomized controlled trials or high quality observational studies (cross-sectional with diagnostic uncertainty and direct comparison of index test results with a reference standard) and at moderate when these types of studies were absent. We then subtracted one point when there was a serious issue identified or two points when there was a very serious issue identified in any of the criteria used to judge the quality of evidence.

^A2^ We assessed study limitations using the QUADAS 2 tool. All studies were cross sectional in design. We downgraded the evidence by 1 point if more than half of all studies did not document blinding, and by 1 point if more than half of studies had unclear or convenience sampling. Three studies evaluated consecutive or random samples and in 1 study sample selection was unclear. One study documented blinding to reference test results, and 3/4 studies were unclear regarding blinding.

^A3^ Uncertainty about directness for false negatives relates to possible detrimental effects from delayed diagnosis of drug resistance. Uncertainty about directness for false positives related to unnecessary us of health care and patient resources through unnecessary administration of second line anti-tuberculous drugs. All studies were conducted on a sample of suspected TB cases at risk for drug resistance. In 1 study, isolates were collected and evaluated from an undescribed population of TB suspects. Of the remaining studies, 2 were conducted in both an inpatient and outpatient setting, and 1 was conducted only in an outpatient setting. Only 1/4 studies was conducted in a high income country limiting generalizability to this setting.

^A4^We assessed heterogeneity by visual inspection of forest plots of accuracy estimates. The sensitivity in the studies varied from 87 to 96% and the specificity varied from 87 to 100%. The variability in sensitivity and specificity is unexplained. However, differing criteria for patient selection and severity of illness of the study populations may have introduced variability in findings among studies. Statistics used to measure heterogeneity in meta-analyses of randomized controlled trials, such as the I-squared statistic, were not considerable suitable for these diagnostic studies.

^A5^ Pooled sensitivity and specificity had narrow confidence intervals

^A6^ We did not restrict our search to English language articles and included manuscripts published in Spanish and French. We did exclude articles published in other languages; in addition we did not include abstracts in our review and could not account for unpublished data. Data included in this review did not allow for formal assessment of publication bias using methods such as funnel plots or regression tests because such techniques have not been found to be helpful for diagnostic test accuracy studies

**Table S2B. Outcome: MTBDR as a replacement test for conventional drug susceptibility testing of rifampicin resistance^A1^**

| **No of Participants (Studies)** | **Study design** | | **Limitations** | **Indirectness** | **Inconsistency** | **Imprecision** | **Publication Bias** | **Quality of Evidence (GRADE)** | **Importance** |
| --- | --- | --- | --- | --- | --- | --- | --- | --- | --- |
| **True positives** | | | | | | | | |  |
| 67 (3) | Cross-sectional | | Very Serious^A2^  (-2) | Serious^A3^  (-1) | No Serious Inconsistency^A4^ | No Serious Imprecision^A5^ | Strongly Suspected^A6^ | Very Low ⊕🌕🌕🌕 | Critical (7-9) |
| **True negatives** | | | | | | | | |  |
| 153 (3) | Cross-sectional | | Very Serious^A2^  (-2) | Serious^A3^  (-1) | No Serious Inconsistency^A4^ | No Serious Imprecision^A5^ | Strongly Suspected^A6^ | Very Low ⊕🌕🌕🌕 | Critical (7-9) |
| **False positives** | | | | | | | | |  |
| 3 (3) | Cross-sectional | | Very Serious^A2^  (-2) | Serious^A3^  (-1) | No Serious Inconsistency^A4^ | No Serious Imprecision^A5^ | Strongly Suspected^A6^ | Very Low ⊕🌕🌕🌕 | Critical (7-9) |
| **False negatives** | | | | | | | | |  |
| 1 (3) | | Cross-sectional | Very Serious^A2^  (-2) | Serious^A3^  (-1) | No Serious Inconsistency^A4^ | No Serious Imprecision^A5^ | Strongly Suspected^A6^ | Very Low ⊕🌕🌕🌕 | Critical (7-9) |

Based on pooled sensitivity = 99% (95% CI 92, 100) and pooled specificity = 98% (95% CI 94, 100)

**Footnotes**

^A1^As recommended, we rated the quality of evidence as high (no points subtracted), moderate (1 point subtracted), low (2 points subtracted), or very low (>2 points subtracted) based on five criteria: imitations, indirectness, inconsistency, imprecision, and publication bias. For each outcome, the quality of evidence started at high when there were randomized controlled trials or high quality observational studies (cross-sectional with diagnostic uncertainty and direct comparison of index test results with a reference standard) and at moderate when these types of studies were absent. We then subtracted one point when there was a serious issue identified or two points when there was a very serious issue identified in any of the criteria used to judge the quality of evidence.

^A2^ We assessed study limitations using the QUADAS 2 tool. All studies were cross sectional in design. We downgraded the evidence by 1 point if more than half of all studies did not document blinding, and by 1 point if more than half of studies had unclear or convenience sampling. In all studies, method of sample selection and blinding strategy were unclear.

^A3^Uncertainty about directness for false negatives relates to possible detrimental effects from delayed diagnosis of drug resistance. Uncertainty about directness for false positives related to unnecessary us of health care and patient resources through unnecessary administration of second line anti-tuberculous drugs. All studies were conducted on a sample of suspected TB cases at risk for drug resistance. In 2 studies, samples were collected and evaluated from an undescribed population of TB suspects. In other study, samples were collected from both an inpatient and outpatient setting. Only 1/3 studies included samples from a low/middle income country limiting generalizability to this setting.

^A4^We assessed heterogeneity by visual inspection of forest plots of accuracy estimates. The sensitivity in the studies varied from 96 to 100% and the specificity varied from 97 to 100%. Estimates of sensitivity and specificity from each study were near perfect. Therefore, we pooled sensitivity and specificity separately as we reasoned that there was little or no correlation between them across studies.

^A5^ Pooled sensitivity and specificity had narrow confidence intervals

^A6^ We did not restrict our search to English language articles and included manuscripts published in Spanish and French. We did exclude articles published in other languages; in addition we did not include abstracts in our review and could not account for unpublished data. Data included in this review did not allow for formal assessment of publication bias using methods such as funnel plots or regression tests because such techniques have not been found to be helpful for diagnostic test accuracy studies

**Table S2C. Outcome: MTBDRplus as a replacement test for conventional drug susceptibility testing of rifampicin resistance^A1^**

| **No of Participants (Studies)** | **Study design** | | **Limitations** | **Indirectness** | **Inconsistency** | **Imprecision** | **Publication Bias** | **Quality of Evidence (GRADE)** | **Importance** |
| --- | --- | --- | --- | --- | --- | --- | --- | --- | --- |
| **True positives** | | | | | | | | |  |
| 1110 (11) | Cross-sectional | | No Serious Limitations^A2^ | Serious^A3^  (-1) | No Serious Inconsistency^A4^ | No Serious Imprecision^A5^ | Strongly Suspected^A6^ | Moderate ⊕⊕⊕🌕 | Critical (7-9) |
| **True negatives** | | | | | | | | |  |
| 2087 (11) | Cross-sectional | | No Serious Limitations^A2^ | Serious^A3^  (-1) | No Serious Inconsistency^A4^ | No Serious Imprecision^A5^ | Strongly Suspected^A6^ | Moderate ⊕⊕⊕🌕 | Critical (7-9) |
| **False positives** | | | | | | | | |  |
| 92 (11) | Cross-sectional | | No Serious Limitations^A2^ | Serious^A3^  (-1) | No Serious Inconsistency^A4^ | No Serious Imprecision^A5^ | Strongly Suspected^A6^ | Moderate ⊕⊕⊕🌕 | Critical (7-9) |
| **False negatives** | | | | | | | | |  |
| 48 (11) | | Cross-sectional | No Serious Limitations^A2^ | Serious^A3^  (-1) | No Serious Inconsistency^A4^ | No Serious Imprecision^A5^ | Strongly Suspected^A6^ | Moderate ⊕⊕⊕🌕 | Critical (7-9) |

Based on pooled sensitivity = 96% (95% CI 95, 97) and pooled specificity = 98% (95% CI 95, 99)

**Footnotes**

^A1^As recommended, we rated the quality of evidence as high (no points subtracted), moderate (1 point subtracted), low (2 points subtracted), or very low (>2 points subtracted) based on five criteria: imitations, indirectness, inconsistency, imprecision, and publication bias. For each outcome, the quality of evidence started at high when there were randomized controlled trials or high quality observational studies (cross-sectional with diagnostic uncertainty and direct comparison of index test results with a reference standard) and at moderate when these types of studies were absent. We then subtracted one point when there was a serious issue identified or two points when there was a very serious issue identified in any of the criteria used to judge the quality of evidence.

^A2^ We assessed study limitations using the QUADAS 2 tool. All studies were cross sectional in design. We downgraded the evidence by 1 point if more than half of all studies did not document blinding, and by 1 point if more than half of studies had unclear or convenience sampling. Eight studies evaluated consecutive or random samples and in 3 studies sample selection was unclear. Ten studies documented blinding to reference test results, and 1 study was unclear regarding blinding. We were also able to perform a subgroup analysis of the studies which collected a random or consecutive sample and blinded researchers to reference test results (n=10). In these studies we found a similar sensitivity (96, 95% CI 94, 97) and specificity (98, 95% CI 93, 99) to pooled estimates.

^A3^Uncertainty about directness for false negatives relates to possible detrimental effects from delayed diagnosis of drug resistance. Uncertainty about directness for false positives related to unnecessary us of health care and patient resources through unnecessary administration of second line anti-tuberculous drugs. All studies were conducted on a sample of suspected TB cases at risk for drug resistance. In 5 studies, isolates were collected and evaluated from an undescribed population of TB suspect. Of the remaining studies, 5 were conducted in both an inpatient and outpatient setting, and 1 was conducted only in an outpatient setting. Only 3 studies included samples from participants in high income countries limiting generalizability to these settings.

^A4^We assessed heterogeneity by visual inspection of forest plots of accuracy estimates. The sensitivity in the studies varied from 60 to 100% and the specificity varied from 78 to 100%. The variability in sensitivity and specificity is unexplained. However, differing criteria for patient selection and severity of illness of the study populations may have introduced variability in findings among studies. Statistics used to measure heterogeneity in meta-analyses of randomized controlled trials, such as the I-squared statistic, were not considerable suitable for these diagnostic studies.

^A5^ Pooled sensitivity and specificity had narrow confidence intervals

^A6^ We did not restrict our search to English language articles and included manuscripts published in Spanish and French. We did exclude articles published in other languages; in addition we did not include abstracts in our review and could not account for unpublished data. Data included in this review did not allow for formal assessment of publication bias using methods such as funnel plots or regression tests because such techniques have not been found to be helpful for diagnostic test accuracy studies

**Table S2D. Outcome: MODS as a replacement test for conventional drug susceptibility testing of rifampicin resistance^A1^**

| **No of Participants (Studies)** | **Study design** | | **Limitations** | **Indirectness** | **Inconsistency** | **Imprecision** | **Publication Bias** | **Quality of Evidence (GRADE)** | **Importance** |
| --- | --- | --- | --- | --- | --- | --- | --- | --- | --- |
| **True positives** | | | | | | | | |  |
| 319 (10) | Cross-sectional | | No Serious Limitations^A2^ | Serious^A3^ (-1) | No Serious Inconsistency^A4^ | No Serious Imprecision^A5^ | Strongly Suspected^A6^ | Moderate ⊕⊕⊕🌕 | Critical (7-9) |
| **True negatives** | | | | | | | | |  |
| 1028 (10) | Cross-sectional | | No Serious Limitations^A2^ | Serious^A3^ (-1) | No Serious Inconsistency^A4^ | No Serious Imprecision^A5^ | Strongly Suspected^A6^ | Moderate ⊕⊕⊕🌕 | Critical (7-9) |
| **False positives** | | | | | | | | |  |
| 37 (10) | Cross-sectional | | No Serious Limitations^A2^ | Serious^A3^ (-1) | No Serious Inconsistency^A4^ | No Serious Imprecision^A5^ | Strongly Suspected^A6^ | Moderate ⊕⊕⊕🌕 | Critical (7-9) |
| **False negatives** | | | | | | | | |  |
| 11 (10) | | Cross-sectional | No Serious Limitations^A2^ | Serious^A3^ (-1) | No Serious Inconsistency^A4^ | No Serious Imprecision^A5^ | Strongly Suspected^A6^ | Moderate ⊕⊕⊕🌕 | Critical (7-9) |

Based on pooled sensitivity = 98% (95% CI 93, 100) and pooled specificity = 99% (95% CI 95, 100)

**Footnotes**

^A1^As recommended, we rated the quality of evidence as high (no points subtracted), moderate (1 point subtracted), low (2 points subtracted), or very low (>2 points subtracted) based on five criteria: imitations, indirectness, inconsistency, imprecision, and publication bias. For each outcome, the quality of evidence started at high when there were randomized controlled trials or high quality observational studies (cross-sectional with diagnostic uncertainty and direct comparison of index test results with a reference standard) and at moderate when these types of studies were absent. We then subtracted one point when there was a serious issue identified or two points when there was a very serious issue identified in any of the criteria used to judge the quality of evidence.

^A2^ We assessed study limitations using the QUADAS 2 tool. All studies were cross sectional in design. We downgraded the evidence by 1 point if more than half of all studies did not document blinding, and by 1 point if more than half of studies had unclear or convenience sampling. Six studies evaluated consecutive or random samples, in 2 studies sample selection was unclear and in 2 studies a convenience sample was selected. Six studies documented blinding to reference test results, 3 studies were unclear regarding blinding, and 1 study did not blind researchers to reference test results.

^A3^Uncertainty about directness for false negatives relates to possible detrimental effects from delayed diagnosis of drug resistance. Uncertainty about directness for false positives related to unnecessary us of health care and patient resources through unnecessary administration of second line anti-tuberculous drugs. All studies were conducted on a sample of suspected TB cases at risk for drug resistance. In 2 studies, isolates were collected and evaluated from an undescribed population of TB suspect. Of the remaining studies, 6 were conducted in both an inpatient and outpatient setting, and 2 were conducted only in a hospital setting. Only 1 study included samples from participants in high income countries limiting generalizability to these settings.

^A4^We assessed heterogeneity by visual inspection of forest plots of accuracy estimates. The sensitivity in the studies varied from 88 to 100% and the specificity varied from 83 to 100%. The variability in sensitivity and specificity is unexplained. However, differing criteria for patient selection and severity of illness of the study populations may have introduced variability in findings among studies. Statistics used to measure heterogeneity in meta-analyses of randomized controlled trials, such as the I-squared statistic, were not considerable suitable for these diagnostic studies.

^A5^ Pooled sensitivity and specificity had narrow confidence intervals

^A6^ We did not restrict our search to English language articles and included manuscripts published in Spanish and French. We did exclude articles published in other languages; in addition we did not include abstracts in our review and could not account for unpublished data. Data included in this review did not allow for formal assessment of publication bias using methods such as funnel plots or regression tests because such techniques have not been found to be helpful for diagnostic test accuracy studies

**Table S2E. Outcome: Nitrate reductase assay (NRA) as a replacement test for conventional drug susceptibility testing of rifampicin resistance^A1^**

| **No of Participants (Studies)** | **Study design** | | **Limitations** | **Indirectness** | **Inconsistency** | **Imprecision** | **Publication Bias** | **Quality of Evidence (GRADE)** | **Importance** |
| --- | --- | --- | --- | --- | --- | --- | --- | --- | --- |
| **True positives** | | | | | | | | |  |
| 672 (19) | Cross-sectional | | Very Serious^A2^  (-2) | Serious^A3^ (-1) | No Serious Inconsistency^A4^ | No Serious Imprecision^A5^ | Strongly Suspected^A6^ | Very Low ⊕🌕🌕🌕 | Critical (7-9) |
| **True negatives** | | | | | | | | |  |
| 1750 (19) | Cross-sectional | | Very Serious^A2^  (-2) | Serious^A3^ (-1) | No Serious Inconsistency^A4^ | No Serious Imprecision^A5^ | Strongly Suspected^A6^ | Very Low ⊕🌕🌕🌕 | Critical (7-9) |
| **False positives** | | | | | | | | |  |
| 9 (19) | Cross-sectional | | Very Serious^A2^  (-2) | Serious^A3^ (-1) | No Serious Inconsistency^A4^ | No Serious Imprecision^A5^ | Strongly Suspected^A6^ | Very Low ⊕🌕🌕🌕 | Critical (7-9) |
| **False negatives** | | | | | | | | |  |
| 18 (19) | | Cross-sectional | Very Serious^A2^  (-2) | Serious^A3^ (-1) | No Serious Inconsistency^A4^ | No Serious Imprecision^A5^ | Strongly Suspected^A6^ | Very Low ⊕🌕🌕🌕 | Critical (7-9) |

Based on pooled sensitivity = 98% (95% CI 96, 99) and pooled specificity = 99% (95% CI 99, 100)

**Footnotes**

^A1^As recommended, we rated the quality of evidence as high (no points subtracted), moderate (1 point subtracted), low (2 points subtracted), or very low (>2 points subtracted) based on five criteria: imitations, indirectness, inconsistency, imprecision, and publication bias. For each outcome, the quality of evidence started at high when there were randomized controlled trials or high quality observational studies (cross-sectional with diagnostic uncertainty and direct comparison of index test results with a reference standard) and at moderate when these types of studies were absent. We then subtracted one point when there was a serious issue identified or two points when there was a very serious issue identified in any of the criteria used to judge the quality of evidence.

^A2^ We assessed study limitations using the QUADAS 2 tool. All studies were cross sectional in design. We downgraded the evidence by 1 point if more than half of all studies did not document blinding, and by 1 point if more than half of studies had unclear or convenience sampling. Six studies documented blinding to reference test results, 12 studies were unclear regarding blinding, and 1 study did not blind researchers to reference test results. Nine studies evaluated consecutive or random samples, and in 10 studies sample selection was unclear. We were able to perform a subgroup analysis of the studies that collected a random or consecutive sample and blinded researchers to reference test results (n=4). In these studies we found a similar sensitivity (97, 95% CI 57, 100) and specificity (99, 95% CI 98, 100) to pooled estimates.

^A3^Uncertainty about directness for false negatives relates to possible detrimental effects from delayed diagnosis of drug resistance. Uncertainty about directness for false positives related to unnecessary us of health care and patient resources through unnecessary administration of second line anti-tuberculous drugs. Eighteen studies were conducted on a sample of suspected TB cases at risk for drug resistance and in one study risk of drug resistance was unclear. In 10 studies, isolates were collected and evaluated from an undescribed population of TB suspects. Of the remaining studies, 8 were conducted in both an inpatient and outpatient setting, and 1 was conducted only in a hospital setting. Only 1 study included samples from participants in a high income country limiting generalizability to this settings.

^A4^We assessed heterogeneity by visual inspection of forest plots of accuracy estimates. The sensitivity in the studies varied from 80 to 100% and the specificity varied from 97 to 100%. The variability in sensitivity is unexplained. However, differing criteria for patient selection and severity of illness of the study populations may have introduced variability in findings among studies. Statistics used to measure heterogeneity in meta-analyses of randomized controlled trials, such as the I-squared statistic, were not considerable suitable for these diagnostic studies.

^A5^ Pooled sensitivity and specificity had narrow confidence intervals.

^A6^ We did not restrict our search to English language articles and included manuscripts published in Spanish and French. We did exclude articles published in other languages; in addition we did not include abstracts in our review and could not account for unpublished data. Data included in this review did not allow for formal assessment of publication bias using methods such as funnel plots or regression tests because such techniques have not been found to be helpful for diagnostic test accuracy studies

**Table S2F. Outcome: Colorimetric redox indicator (CRI) assays as a replacement test for conventional drug susceptibility testing of rifampicin resistance^A1^**

| **No of Participants (Studies)** | **Study design** | | **Limitations** | **Indirectness** | **Inconsistency** | **Imprecision** | **Publication Bias** | **Quality of Evidence (GRADE)** | **Importance** |
| --- | --- | --- | --- | --- | --- | --- | --- | --- | --- |
| **True positives** | | | | | | | | |  |
| 444 (14) | Cross-sectional | | Very Serious^A2^  (-2) | Serious^A3^  (-1) | No Serious Inconsistency^A4^ | No Serious Imprecision^A5^ | Strongly Suspected^A6^ | Very Low ⊕🌕🌕🌕 | Critical (7-9) |
| **True negatives** | | | | | | | | |  |
| 1173 (14) | Cross-sectional | | Very Serious^A2^  (-2) | Serious^A3^  (-1) | No Serious Inconsistency^A4^ | No Serious Imprecision^A5^ | Strongly Suspected^A6^ | Very Low ⊕🌕🌕🌕 | Critical (7-9) |
| **False positives** | | | | | | | | |  |
| 3 (14) | Cross-sectional | | Very Serious^A2^  (-2) | Serious^A3^  (-1) | No Serious Inconsistency^A4^ | No Serious Imprecision^A5^ | Strongly Suspected^A6^ | Very Low ⊕🌕🌕🌕 | Critical (7-9) |
| **False negatives** | | | | | | | | |  |
| 9 (14) | | Cross-sectional | Very Serious^A2^  (-2) | Serious^A3^  (-1) | No Serious Inconsistency^A4^ | No Serious Imprecision^A5^ | Strongly Suspected^A6^ | Very Low ⊕🌕🌕🌕 | Critical (7-9) |

Based on pooled sensitivity = 99% (95% CI 96, 100) and pooled specificity = 99% (95% CI 99, 100)

**Footnotes**

^A1^As recommended, we rated the quality of evidence as high (no points subtracted), moderate (1 point subtracted), low (2 points subtracted), or very low (>2 points subtracted) based on five criteria: imitations, indirectness, inconsistency, imprecision, and publication bias. For each outcome, the quality of evidence started at high when there were randomized controlled trials or high quality observational studies (cross-sectional with diagnostic uncertainty and direct comparison of index test results with a reference standard) and at moderate when these types of studies were absent. We then subtracted one point when there was a serious issue identified or two points when there was a very serious issue identified in any of the criteria used to judge the quality of evidence.

^A2^ We assessed study limitations using the QUADAS 2 tool. All studies were cross sectional in design. We downgraded the evidence by 1 point if more than half of all studies did not document blinding, and by 1 point if more than half of studies had unclear or convenience sampling. Two studies evaluated consecutive or random samples, in 11 studies sample selection was unclear, and 1 study evaluated a convenience sample. Three studies documented blinding to reference test results, 9 studies were unclear regarding blinding, and 2 studies did not blind researchers to reference test results.

Ten studies were conducted on a sample of suspected TB cases at risk for drug resistance and in 4 studies the risk of drug resistance was unclear. All studies were cross-sectional in design.

^A3^Uncertainty about directness for false negatives relates to possible detrimental effects from delayed diagnosis of drug resistance. Uncertainty about directness for false positives related to unnecessary us of health care and patient resources through unnecessary administration of second line anti-tuberculous drugs. Ten studies were conducted on a sample of suspected TB cases at risk for drug resistance and in 4 studies the risk of drug resistance was unclear. In 11 studies, isolates were collected and evaluated from an undescribed population of TB suspects. Of the remaining studies, 2 were conducted in both an inpatient and outpatient setting, and 1 was conducted only in a hospital setting.

^A4^We assessed heterogeneity by visual inspection of forest plots of accuracy estimates. The sensitivity in the studies varied from 87 to 100% and the specificity varied from 96 to 100%. The variability in sensitivity is unexplained. However, differing criteria for patient selection and severity of illness of the study populations may have introduced variability in findings among studies. Statistics used to measure heterogeneity in meta-analyses of randomized controlled trials, such as the I-squared statistic, were not considerable suitable for these diagnostic studies.

^A5^ Pooled sensitivity and specificity had narrow confidence intervals

^A6^ We did not restrict our search to English language articles and included manuscripts published in Spanish and French. We did exclude articles published in other languages, in addition we did not include abstracts in our review and could not account for unpublished data. Data included in this review did not allow for formal assessment of publication bias using methods such as funnel plots or regression tests because such techniques have not been found to be helpful for diagnostic test accuracy studies

**Table S3. GRADE Summary of Findings**

| Review question: What is the diagnostic accuracy of rapid tests for detection of resistance to rifampicin?  Patients/population: Patients who are suspected of having TB  Setting: Clinical centers evaluating TB suspects for rifampicin resistance  Index tests: WHO-endorsed genotypic and phenotypic tests  Importance: A rapid, accurate, simple test could replace conventional culture and DST and expand testing to lower levels of the health service  Reference standard: Conventional drug susceptibility testing (DST) by solid or liquid culture  Studies: Cross-sectional | | | | | | |
| --- | --- | --- | --- | --- | --- | --- |
| **Outcomes: TP, TN, FP, FN** | **Effect %**  **(95% CI)** | **No. of Participants (Studies)** | **What do these results mean given a 3% prevalence of rifampicin resistance among suspects being screened for TB?** | **What do these results mean given a 15% prevalence of rifampicin resistance among suspects being screened for TB?** | **What do these results mean given a 30% prevalence of rifampicin resistance among suspects being screened for TB?** | **Quality of Evidence** |
| **Diagnostic accuracy for rifampicin resistance** |
| INNO-LiPA Rif.TB | Pooled sensitivity  94% (87, 98)  Pooled specificity  99% (94, 100) | 947 (4) | With a prevalence of 3%, 30/1000 will have rifampicin resistance. Of these, 28 (TP) will be identified; 2 (FN) will be missed by a commercial test. Of the 970 patients without TB, 958 (TN) will not be treated; 12 (FP) will be unnecessarily treated | With a prevalence of 15%, 150/1000 will have rifampicin resistance. Of these, 141 (TP) will be identified; 9 (FN) will be missed by a commercial test. Of the 850 patients without TB, 839 (TN) will not be treated; 11 (FP) will be unnecessarily treated | With a prevalence of 30%, 300/1000 will have TB. Of these, 282 (TP) will be identified; 18 (FN) will be missed by a commercial test. Of the 700 patients without TB, 691 (TN) will not be treated; 9 (FP) will be unnecessarily treated | Low  ⊕⊕🌕🌕 |
| MTBDR | Pooled sensitivity  99% (92, 100)  Pooled specificity  98% (94, 100) | 224 (3) | With a prevalence of 3%, 30/1000 will have rifampicin resistance. Of these, 29 (TP) will be identified; 1 (FN) will be missed by a commercial test. Of the 970 patients without TB, 950 (TN) will not be treated; 20 (FP) will be unnecessarily treated | With a prevalence of 15%, 150/1000 will have rifampicin resistance. Of these, 148 (TP) will be identified; 2 (FN) will be missed by a commercial test. Of the 850 patients without TB, 833 (TN) will not be treated; 17 (FP) will be unnecessarily treated | With a prevalence of 30%, 300/1000 will have TB. Of these, 297 (TP) will be identified; 3 (FN) will be missed by a commercial test. Of the 700 patients without TB, 686 (TN) will not be treated; 14 (FP) will be unnecessarily treated | Very Low ⊕🌕🌕🌕 |
| MTBDR plus | Pooled sensitivity  96% (95, 97) Pooled specificity  98% (95, 99) | 3337 (11) | With a prevalence of 3%, 30/1000 will have rifampicin resistance. Of these, 29 (TP) will be identified; 1 (FN) will be missed by a commercial test. Of the 970 patients without TB, 949 (TN) will not be treated; 21 (FP) will be unnecessarily treated | With a prevalence of 15%, 150/1000 will have rifampicin resistance. Of these, 144 (TP) will be identified; 6 (FN) will be missed by a commercial test. Of the 850 patients without TB, 832 (TN) will not be treated; 18 (FP) will be unnecessarily treated | With a prevalence of 30%, 300/1000 will have TB. Of these, 288 (TP) will be identified; 12 (FN) will be missed by a commercial test. Of the 700 patients without TB, 685 (TN) will not be treated; 15 (FP) will be unnecessarily treated | Moderate ⊕⊕⊕🌕 |
| MODS | Pooled sensitivity  98% (93, 100) Pooled specificity  99% (95, 100) | 1395 (10) | With a prevalence of 3%, 30/1000 will have rifampicin resistance. Of these, 29 (TP) will be identified; 1 (FN) will be missed by a commercial test. Of the 970 patients without TB, 962 (TN) will not be treated; 8 (FP) will be unnecessarily treated | With a prevalence of 15%, 150/1000 will have rifampicin resistance. Of these, 147 (TP) will be identified; 3 (FN) will be missed by a commercial test. Of the 850 patients without TB, 843 (TN) will not be treated; 7 (FP) will be unnecessarily treated | With a prevalence of 30%, 300/1000 will have TB. Of these, 294 (TP) will be identified; 6 (FN) will be missed by a commercial test. Of the 700 patients without TB, 695 (TN) will not be treated; 5 (FP) will be unnecessarily treated | Moderate ⊕⊕⊕🌕 |
| Nitrate Reductase Assay (NRA) | Pooled sensitivity  98% (96, 99) Pooled specificity  99% (99, 100) | 2289 (19) | With a prevalence of 3%, 30/1000 will have rifampicin resistance. Of these, 29 (TP) will be identified; 1 (FN) will be missed by a commercial test. Of the 970 patients without TB, 968 (TN) will not be treated; 2 (FP) will be unnecessarily treated | With a prevalence of 15%, 150/1000 will have rifampicin resistance. Of these, 147 (TP) will be identified; 3 (FN) will be missed by a commercial test. Of the 850 patients without TB, 848 (TN) will not be treated; 2 (FP) will be unnecessarily treated | With a prevalence of 30%, 300/1000 will have TB. Of these, 293 (TP) will be identified; 7 (FN) will be missed by a commercial test. Of the 700 patients without TB, 699 (TN) will not be treated; 1 (FP) will be unnecessarily treated | Very Low ⊕🌕🌕🌕 |
| Colorimetric Redox Indicator (CRI) Assays | Pooled sensitivity  99% (96, 100) Pooled specificity  99% (99, 100) | 1629 (14) | With a prevalence of 3%, 30/1000 will have rifampicin resistance. Of these, 29 (TP) will be identified; 1 (FN) will be missed by a commercial test. Of the 970 patients without TB, 968 (TN) will not be treated; 2 (FP) will be unnecessarily treated | With a prevalence of 15%, 150/1000 will have rifampicin resistance. Of these, 148 (TP) will be identified; 2 (FN) will be missed by a commercial test. Of the 850 patients without TB, 848 (TN) will not be treated; 2 (FP) will be unnecessarily treated | With a prevalence of 30%, 300/1000 will have TB. Of these, 296 (TP) will be identified; 4 (FN) will be missed by a commercial test. Of the 700 patients without TB, 698 (TN) will not be treated; 2 (FP) will be unnecessarily treated | Very Low ⊕🌕🌕🌕 |

TP, true positive; FN, false negative; TN, true negative; FP, false positive

**Figure S1. Assessment of study quality.** For each QUADAS item, two reviewers independently determined whether a study did or did not meet the quality criterion, or whether it was unclear. The percentage of studies meeting each relevant QUADAS item are shown

| **Study** | **RISK OF BIAS** | | | | **APPLICABILITY CONCERNS** | | |
| --- | --- | --- | --- | --- | --- | --- | --- |
|  | **PATIENT SELECTION** | **INDEX TEST** | **REFERENCE STANDARD** | **FLOW AND TIMING** | **PATIENT SELECTION** | **INDEX TEST** | **REFERENCE STANDARD** |
| Affolabi 2007 | ? | ☺ | ☺ | ☺ | ☺ | ☺ | ☺ |
| Affolabi I 2008 | ☺ | ☺ | ☺ | ☺ | ☺ | ☺ | ☺ |
| Affolabi II 2008 | ☺ | ☺ | ☺ | ☺ | ☺ | ☺ | ☺ |
| Ahmad 2009 | ☹ | ? | ☺ | ☺ | ? | ☺ | ☺ |
| Albert 2010 | ☺ | ☺ | ☺ | ☺ | ☺ | ☺ | ☺ |
| Anek 2010 | ? | ☺ | ☺ | ☺ | ☺ | ☺ | ☺ |
| Ani 2009 | ? | ? | ? | ☺ | ☺ | ☺ | ☺ |
| Asencios 2008 | ☺ | ? | ? | ☺ | ☺ | ☺ | ☺ |
| Banfi 2003 | ? | ? | ? | ☺ | ☺ | ☺ | ☺ |
| Barnard 2008 | ☺ | ☺ | ☺ | ☺ | ☺ | ☺ | ☺ |
| Bwanga 2011 | ☺ | ? | ? | ☺ | ☺ | ☺ | ☺ |
| Chauca 2007 | ? | ? | ? | ☺ | ☺ | ☺ | ☺ |
| Cirillo 2004 | ? | ? | ☺ | ☺ | ☺ | ☺ | ☺ |
| Coban 2004 | ? | ? | ? | ☺ | ☺ | ☺ | ☺ |
| De la Iglesia 2009 | ? | ☺ | ☺ | ☺ | ? | ☺ | ☺ |
| Duo 2011 | ? | ☺ | ☺ | ☺ | ☺ | ☺ | ☺ |
| Ejigu 2008 | ☹ | ☺ | ☺ | ☺ | ☺ | ☺ | ☺ |
| Evans 2009 | ? | ☺ | ☺ | ☺ | ☺ | ☺ | ☺ |
| Franzblau 1998 | ☹ | ? | ☺ | ☺ | ☺ | ☺ | ☺ |
| Giacomazzi 2010 | ? | ? | ? | ☺ | ☺ | ☺ | ☺ |
| Gitti 2011 | ☺ | ☺ | ☺ | ☺ | ☺ | ☺ | ☺ |
| Gupta 2010 | ? | ? | ? | ☺ | ☺ | ☺ | ☺ |
| Gupta 2011 | ? | ? | ? | ☺ | ☺ | ☺ | ☺ |
| Hillemann 2006 | ☹ | ? | ? | ☺ | ☺ | ☺ | ☺ |
| Huang 2009 | ? | ☺ | ? | ☺ | ☺ | ☺ | ☺ |
| Huyen 2010 | ☺ | ☺ | ? | ☺ | ☺ | ☺ | ☺ |
| Lacoma 2008 | ? | ☺ | ☺ | ☺ | ? | ☺ | ☺ |
| Limaye 2010 | ☺ | ☺ | ☺ | ☺ | ☺ | ☺ | ☺ |
| Luna-Herrera 2003 | ? | ? | ? | ☺ | ☺ | ☺ | ☺ |
| Makinen 2006 | ? | ? | ? | ☺ | ☺ | ☺ | ☺ |
| Mello 2007 | ☺ | ☺ | ☺ | ☺ | ☺ | ☺ | ☺ |
| Mendoza 2010 | ☺ | ? | ? | ☺ | ☺ | ☺ | ☺ |
| Mengatto 2006 | ? | ☺ | ☺ | ☺ | ? | ☺ | ☺ |
| Mironova 2011 | ☺ | ? | ? | ☺ | ☺ | ☺ | ☺ |
| Miyata 2011 | ? | ? | ☺ | ☺ | ☺ | ☹ | ☺ |
| Moore 2006 | ☺ | ☺ | ☺ | ☺ | ☺ | ☺ | ☺ |
| Morcillo 2010 | ? | ? | ☺ | ☺ | ☺ | ☺ | ☺ |
| Musa 2005 | ☺ | ? | ? | ☺ | ☺ | ☺ | ☺ |
| Nateche 2006 | ? | ? | ? | ☺ | ? | ☺ | ☺ |
| Ogwang 2009 | ☺ | ☺ | ☺ | ☺ | ☺ | ☺ | ☺ |
| Palomino 2002 | ? | ? | ? | ☺ | ? | ☺ | ☺ |
| Palomino 1999 | ☹ | ? | ? | ☺ | ☺ | ☺ | ☺ |
| Park 2002 | ☹ | ☺ | ☺ | ☺ | ☺ | ☺ | ☺ |
| Pontino 2006 | ? | ? | ? | ☺ | ☺ | ☺ | ☺ |
| Raut 2008 | ☺ | ? | ? | ☺ | ☺ | ☺ | ☺ |
| Reis 2004 | ? | ? | ? | ☺ | ? | ☺ | ☺ |
| Rigouts 2011 | ☺ | ☺ | ☺ | ☺ | ☺ | ☺ | ☺ |
| Rosales 2011 | ☺ | ☺ | ☺ | ☺ | ☺ | ☺ | ☺ |
| Sam 20006 | ☺ | ? | ? | ☺ | ☺ | ☺ | ? |
| Scott 2011 | ☺ | ? | ☺ | ☺ | ☺ | ☺ | ☺ |
| Sethi 2004 | ☺ | ? | ? | ☺ | ☺ | ☺ | ☺ |
| Shah 2011 | ☺ | ? | ? | ☺ | ☺ | ☺ | ☺ |
| Shiferaw 2007 | ☺ | ? | ? | ☺ | ☺ | ☺ | ☺ |
| Shikama Mde 2009 | ? | ? | ? | ☺ | ☺ | ☺ | ☺ |
| Shikama 2009 | ? | ? | ? | ☺ | ? | ☺ | ☺ |
| Skenders 2011 | ☺ | ? | ☺ | ☺ | ☺ | ☺ | ☺ |
| Solis 2005 | ☺ | ? | ? | ☺ | ☺ | ☺ | ☺ |
| Somoskovi 2006 | ? | ? | ☺ | ☺ | ☺ | ☺ | ☺ |
| Visalakshiu 2010 | ? | ? | ? | ☺ | ☺ | ☺ | ☺ |
|  |  |  |  |  |  |  |  |

☺Low Risk ☹High Risk ? Unclear Risk

**Figure S2 Forest plot: Sensitivity and Specificity of the INNO LiPA. The forest plot displays sensitivity and specificity results for individual studies. Letters after the study year designate different subgroups in the same paper.**

**
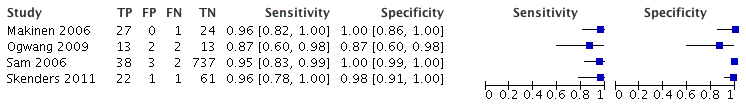
**

**Figure S3 Forest plot: Sensitivity and Specificity of the MTBDR assay. The forest plot displays sensitivity and specificity results for individual studies. Letters after the study year designate different subgroups in the same paper.**

**
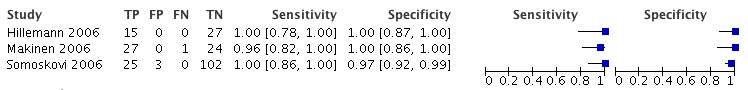
**

**Figure S4 Forest plot: Sensitivity and Specificity of the MTBDRplus assay. The forest plot displays sensitivity and specificity results for individual studies. Letters after the study year designate different subgroups in the same paper.**

**
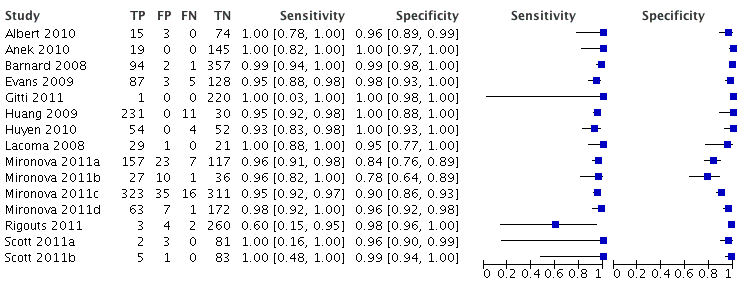
**

**Figure S5 Forest plot: Sensitivity and Specificity of the MODS assay. The forest plot displays sensitivity and specificity results for individual studies. Letters after the study year designate different subgroups in the same paper.**

**
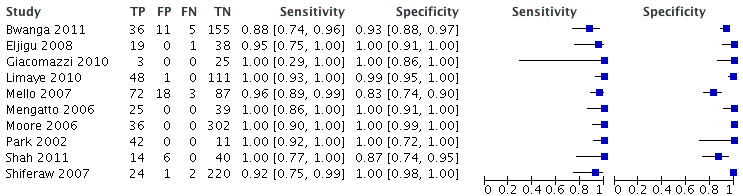
**

**Figure S6 Forest plot: Sensitivity and Specificity of the Nitrate Reductase assay. The forest plot displays sensitivity and specificity results for individual studies. Letters after the study year designate different subgroups in the same paper.**

**
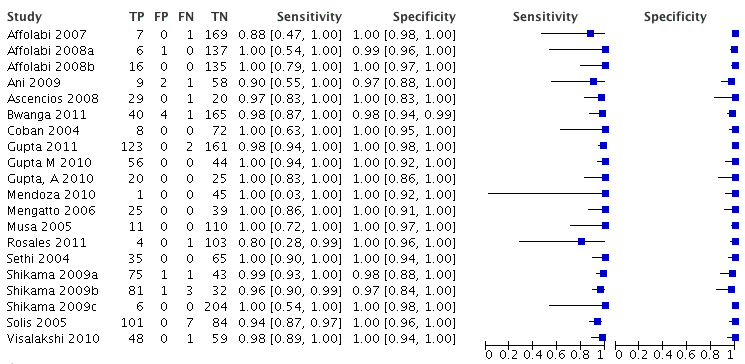
**

**Figure S7 Forest plot: Sensitivity and Specificity of the CRI assay. The forest plot displays sensitivity and specificity results for individual studies. Letters after the study year designate different subgroups in the same paper.**

**
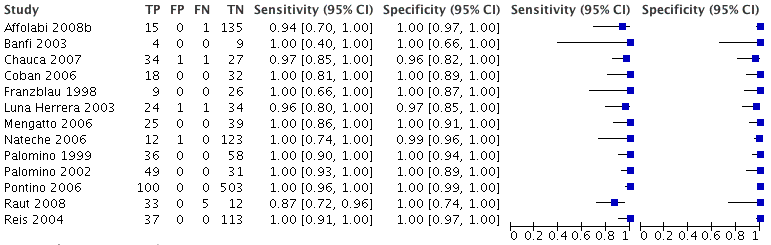
**
