# Supplementary material for: Systematic Review of the Performance of Rapid Rifampicin Resistance Testing for Drug-Resistant Tuberculosis
Source: PLoS One. 2013 Oct 3;8(10):e76533. doi: 10.1371/journal.pone.0076533 (PMC3789679; doi:10.1371/journal.pone.0076533)
Supplement: File S2 — Appendix S1, Search strategy. Appendix S2, Characteristics of included studies. Appendix S3, Methodological Quality Assessment. QUADAS 2 evaluation. Appendix S4, references for included and excluded studies. (DOC) [file pone.0076533.s003.doc]

**APPENDIX– SUPPLEMENTAL METHODS**

**METHODS**

**Appendix S1: Search strategy.**

**Detailed search strategies for Medline and Embase, Performed on January 1^st^, 2012**

| **Search set** | **Pubmed** | **Embase (OVID)** |
| --- | --- | --- |
| 1 | Tuberculosis ti, ab | tuberculosis.mp. or drug resistant tuberculosis/ or lung tuberculosis/ or multidrug resistant tuberculosis/ or Mycobacterium tuberculosis/ |
| 2 | Tuberculosis, Pulmonary [MeSH] | (TB or MDR-TB).mp. |
| 3 | Tuberculosis, Multidrug-Resistant [MeSH] | (rifampicin resist* and (tuberculosis or TB)).mp |
| 4 | Mycobacterium tuberculosis [Mesh] | (rifampin resist* and (TB or tuberculosis)).mp |
| 5 | TB ti, ab | 1 or 2 or 3 or 4 |
| 6 | MDR-TB ti, ab | (line probe and (test* or assay*)).mp |
| 7 | (Multidrug-resistan* tuberculo*) ti, ab | liPA.mp. |
| 8 | Rifampicin resist* ti, ab | INNO-LIPA.mp |
| 9 | Rifampin resist* ti, ab | microscopic-observation drug susceptibility.mp. |
| 10 | 1-9/OR | MODS.mp. |
| 11 | (Line probe) AND (test* OR assay*) ti, ab | (nitrate reductase or NRA).mp. |
| 12 | LiPA ti, ab | griess.mp. |
| 13 | INNO-LiPA ti, ab | colorimetric redox indicator.mp. |
| 14 | microscopic-observation drug-susceptibility ti, ab | *colorimetry/ |
| 15 | MODS ti, ab | (CRI or Alamar blue or tetrazolium bromide or resazurin).mp. |
| 16 | Nitrate reductase ti, ab, MeSH |  |
| 17 | NRA ti, ab | genotype MTBDR*. |
| 18 | Griess ti, ab | direct susceptibility.mp. |
| 19 | colorimetric redox indicator ti, ab | DST.mp. |
| 20 | Colorimetry/methods [MeSH] | 6 or 7 or 8 or 9 or 10 or 11 or 12 or 13 or 14 or 15 or 16 or 17 or 18 or 19 |
| 21 | CRI ti, ab | 5 and 20 |
| 22 | Alamar blue ti, ab | limit 21 to yr="2006 -Current" |
| 23 | Tetrazolium bromide ti, ab |  |
| 24 | Resazurin ti, ab |  |
| 25 | Genotype MTBDR* ti, ab |  |
| 26 | Direct susceptibility ti, ab |  |
| 27 | DST ti, ab |  |
| 28 | 11 or 12 or 13 or 14 or 15 or 16 or 17 or 18 or 19 or 20 or 21 or 22 or 23 or 24 or 25 or 26 or 27 |  |
| 29 | 10 AND 28 |  |
| 30 | Limits: Publication Date from 2006/01 |  |
|  |  |  |

**Appendix S2:** Characteristics of included studies

| **Study Author** | **Year of Publication** | **Country of Participants** | **Study Design** | **Reference Standard** | **Type of Specimen** | **Smear Positive** | **Retreatment Cases** | **Total Number of Participants** |
| --- | --- | --- | --- | --- | --- | --- | --- | --- |
| **INNO-LiPA Rif.TB** | | | | | | | | |
| Makinen | 2006 | Finland | Cross-sectional | 7H10 Proportion method | Indirect | Unclear | Unclear | 52 |
| Ogwang | 2009 | Uganda | Cross-sectional | BACTEC 460 | Direct | 100% | 100% | 30 |
| Sam | 2006 | United Kingdom | Cross-sectional | LJ Resistance ratio method | Direct | 96% | Unclear | 780 |
| Skenders | 2011 | Latvia | Cross-sectional | LJ Proportion method | Direct | 100% | Unclear | 85 |
| **GenoType® MTBDR** | | | | | | | | |
| Hillemann | 2006 | Germany, Azerbaijan, and Uzbekistan | Cross-sectional | LJ Proportion method and MGIT 960 | Direct | 100% | Unclear | 42 |
| Makinen | 2006 | Finland | Cross-sectional | 7H10 Proportion method | Indirect | Unclear | Unclear | 52 |
| Somoskovi | 2006 | USA | Cross-sectional | BACTEC 460 | Direct | 100% | Unclear | 130 |
| **GenoType® MTBDR Plus** | | | | | | | | |
| Albert | 2010 | Uganda | Cross-sectional | MGIT 960 | Direct | 100% | 100% | 92 |
| Anek- Vorapong | 2010 | Thailand | Cross-sectional | MGIT 960 | Direct | 100% | Unclear | 164 |
| Barnard | 2008 | South Africa | Cross-sectional | MGIT 960 | Direct | 100% | Unclear | 454 |
| Evans | 2009 | South Africa | Cross-sectional | MGIT 960 | Indirect | Unclear | Unclear | 223 |
| Gitti | 2011 | Greece | Cross-sectional | LJ Proportion method | Indirect | Unclear | Unclear | 221 |
| Huang | 2009 | Taiwan | Cross-sectional | 7H10/11 Proportion method or MGIT 960 | Indirect | Unclear | Unclear | 272 |
| Huyen | 2010 | Vietnam | Cross-sectional | LJ Proportion method | Indirect | 100% | 50% | 110 |
| Lacoma | 2008 | Spain | Cross-sectional | BACTEC 460 | Direct and indirect | 49% | Unclear | 51 |
| Mironova | 2011a | Estonia, Latvia, Lithuania, and Russia | Cross-sectional | LJ Proportion method | Direct | 100% | Unclear | 304 |
| Mironova | 2011b | Estonia, Latvia, Lithuania, and Russia | Cross-sectional | BACTEC 460 or LJ Proportion method | Direct and indirect | Unclear | Unclear | 1002 |
| Rigouts | 2011 | Tanzania | Cross-sectional | LJ Proportion method | Direct | 100% | Unclear | 269 |
| Scott | 2011 | South Africa | Cross-sectional | MGIT 960 | Direct and indirect | 73% | Unclear | 175 |
| **MODS** | | | | | | | | |
| Bwanga | 2011 | Uganda | Cross-sectional | LJ Proportion method | Direct | 100% | 100% | 207 |
| Eljigu | 2008 | Ethiopia | Cross-sectional | BACTEC 460 | Direct | 100% | 40% | 58 |
| Giacomazzi | 2010 | Ecuador | Cross-sectional | LJ Proportion method | Direct | Unclear | Unclear | 28 |
| Limaye | 2010 | India | Cross-sectional | LJ Proportion method | Direct | 100% | Unclear | 160 |
| Mello | 2007 | Brazil and Honduras | Cross-sectional | LJ Proportion method | Direct | 100% | Unclear | 180 |
| Mengatto | 2006 | Argentina | Cross-sectional | LJ Proportion method | Indirect | Unclear | Unclear | 64 |
| Moore | 2006 | Peru | Cross-sectional | LJ Proportion method | Direct | 55% | Unclear | 338 |
| Park | 2002 | USA | Cross-sectional | 7H10 Proportion method | Indirect | Unclear | Unclear | 53 |
| Shah | 2011 | South Africa | Cross-sectional | 7H10 Proportion method | Direct | 56% | 25% | 60 |
| Shiferaw | 2007 | Ethiopia | Cross-sectional | 7H10 Proportion method | Direct | 100% | Unclear | 247 |
| **Nitrate Reductase Assay** | | | | | | | | |
| Affolabi | 2007 | Benin | Cross-sectional | LJ Proportion method | Direct | 100% | Unclear | 177 |
| Affolabi | 2008a | Benin | Cross-sectional | LJ Proportion method | Direct | 100% | Unclear | 144 |
| Affolabi | 2008b | Benin | Cross-sectional | LJ Proportion method | Indirect | Unclear | Unclear | 151 |
| Ani | 2009 | Nigeria | Cross-sectional | LJ Proportion method | Indirect | Unclear | Unclear | 70 |
| Ascencios | 2008 | Peru | Cross-sectional | 7H10 Proportion method | Direct | 100% | Unclear | 50 |
| Bwanga | 2011 | Uganda | Cross-sectional | LJ Proportion method | Direct | 100% | 100% | 210 |
| Coban | 2004 | Turkey | Cross-sectional | LJ Proportion method | Indirect | Unclear | Unclear | 80 |
| Gupta, A | 2010 | India | Cross-sectional | LJ Proportion method | Direct | 100% | Unclear | 45 |
| Gupta, A | 2011 | India | Cross-sectional | LJ Proportion method | Indirect | Unclear | Unclear | 286 |
| Gupta, M | 2010 | India | Cross-sectional | LJ Proportion method | Direct | 100% | Unclear | 100 |
| Mendoza | 2010 | Venezuela | Cross-sectional | LJ Proportion method | Indirect | Unclear | Unclear | 46 |
| Mengatto | 2006 | Argentina | Cross-sectional | LJ Proportion method | Indirect | Unclear | Unclear | 64 |
| Musa | 2005 | Argentina | Cross-sectional | LJ Proportion method | Direct | 100% | Unclear | 121 |
| Rosales | 2011 | Honduras | Cross-sectional | LJ Proportion method | Direct | 100% | 38% | 108 |
| Sethi | 2004 | India | Cross-sectional | LJ Proportion method | Indirect | Unclear | Unclear | 100 |
| Shikama | 2009a | Brazil | Cross-sectional | LJ Proportion method | Direct | 100% | Unclear | 210 |
| Shikama | 2009b | Brazil | Cross-sectional | LJ Proportion method | Indirect | Unclear | Unclear | 120 |
| Solis | 2005 | Peru | Cross-sectional | LJ Proportion method | Direct | 100% | Unclear | 192 |
| Visalakshi | 2010 | India | Cross-sectional | LJ Proportion method | Direct | 100% | Unclear | 108 |
| **Colorimetric Redox Indicator Assays** | | | | | | | | |
| **Alamar Blue** | | | | | | | | |
| Chauca | 2007 | Peru | Cross-sectional | LJ Proportion method | Indirect | Unclear | Unclear | 63 |
| Franzblau | 1998 | USA | Cross-sectional | BACTEC 460 | Indirect | Unclear | Unclear | 35 |
| Luna-Herrara | 2003 | Mexico | Cross-sectional | 7H10 Proportion method | Indirect | Unclear | Unclear | 60 |
| Palomino | 1999 | Belgium | Cross-sectional | LJ Proportion method | Indirect | Unclear | Unclear | 94 |
| Reis | 2004 | Brazil | Cross-sectional | LJ Proportion method | Indirect | Unclear | Unclear | 150 |
| **Resazurin** | | | | | | | | |
| Affolabi | 2008 | Benin | Cross-sectional | LJ Proportion method | Indirect | Unclear | Unclear | 151 |
| Banfi | 2003 | Italy | Cross-sectional | 7H11 Proportion method | Indirect | Unclear | Unclear | 13 |
| Coban | 2006 | Turkey | Cross-sectional | BACTEC 460 | Indirect | Unclear | Unclear | 50 |
| Miyata | 2011 | Brazil | Cross-sectional | MGIT 960 | Indirect | Unclear | Unclear | 80 |
| Nateche | 2006 | Algeria | Cross-sectional | LJ Proportion method | Indirect | Unclear | Unclear | 136 |
| Palomino | 2002 | Peru and Bolivia | Cross-sectional | LJ Proportion method | Indirect | Unclear | Unclear | 80 |
| **Tetrazolium** | | | | | | | | |
| Mengatto | 2006 | Argentina | Cross-sectional | LJ Proportion method | Indirect | Unclear | Unclear | 64 |
| Pontino | 2006 | Argentina | Cross-sectional | LJ Proportion method | Indirect | Unclear | Unclear | 603 |
| Raut | 2008 | India | Cross-sectional | LJ Proportion method | Indirect | Unclear | Unclear | 50 |

**Appendix S3: Methodological Quality Assessment. QUADAS 2 evaluation**

| **Quality Assessment –QUADAS 2 The final score is High/Low/Unclear** | | | |
| --- | --- | --- | --- |
| **Domain 1 Patient Selection** | | | |
| *Risk of Bias* | | | |
| Notes (use if helpful): Describe methods of patients selection: | | | |
| 1. Was a consecutive or random sample of patients enrolled? | Yes | No | Unclear |
| 2. Was a case-control design avoided?  Case-control studies are an exclusion criterion for this review | Yes | No | Unclear |
| Could the study have introduced bias? | High | Low | Unclear |
| Score High Risk of Bias for the following answer:  1. No  Score Low Risk of Bias for the following answers:  1. Yes  Score Unclear Risk of Bias for the following answer  1. Unclear | | | |
| *Applicability* (describe included patients: prior testing, presentation, intended use, setting)  Are there concerns that included patients do not match review question?  1. High Concern about applicability is not likely in this group of studies  2. Score Low Concern about applicability in most studies, in particular if  TB suspects or  MDR suspects  - patients with history of TB or TB treatment  - patients on TB treatment for pulmonary TB w/o sputum conversion  - previously treated TB suspects who are smear+  3. Score Unclear concern about applicability if your judgment is not clearly 1 or 2 and for  - TB isolates with no information about patients  ­- Setting was reference laboratory | High | Low | Unclear |
| **Domain 2 Index test** | | | |
| *Risk of Bias* | | | |
| Notes (use if helpful): Describe the index test and how it was conducted and interpreted | | | |
| 1. Were the index test results interpreted without knowledge of the results of the reference standard? (Blinding needs to be stated for a yes answer unless the index test result was clearly available before the ref standard result was available. Do not assume these tests are being done at the same time unless clearly stated) | Yes | No | Unclear |
| 2. If a threshold (cut-point) was used, was it pre-specified? This will usually be ‘yes’ unless study looks at test development/refinement. If a commercial test, such as line probe assay, MGIT, is used according manufacturers’ directions, score as Yes | Yes | No | Unclear |
| Could the conduct or interpretation of the index test have introduced bias? | High | Low | Unclear |
| Score High Risk of Bias for the following answers:  1. No/2. No  Score Low Risk of Bias for the following answer:  1. Yes/2.Yes  Score Unclear Risk of Bias for all other answers | | | |
| *Applicability*  Are there concerns that the index test, its conduct, or interpretation differ from the review question?  If threshold is NOT pre-specified score, HIGH CONCERN  If threshold is pre-specified score, LOW CONCERN  if threshold is UNCLEAR, score UNCLEAR CONCERN | High | Low | Unclear |
| **Domain 3 Reference standard (In this review, all criteria are met by all studies)** | | | |
| *Risk of Bias* | | | |
| Notes (use if helpful): Describe reference standard and how it was conducted and interpreted | | | |
| 1. Is the reference standard likely to correctly classify the target condition? | Yes | No | Unclear |
| 2. Were the reference standard results interpreted without knowledge of the results of the index test? (We assume solid and liquid culture are objective in interpretation, the answer is Yes for all studies) | Yes | No | Unclear |
| Could the reference standard, its conduct or its interpretation have introduced bias? | High | Low | Unclear |
| *Applicability*  Are there concerns that the target condition as defined by the reference standard does not match the review question? | High | Low | Unclear |
| **Domain 4 Flow and Timing** | | | |
| *Risk of Bias* |  |  |  |
| Describe any patients who did not receive the index test and /or reference standard and were excluded from the 2 x 2 table (refer to flow diagram) | | | |
| 1. Was there an appropriate interval between index test and reference standard? | Yes | No | Unclear |
| 2. Did all patients receive a reference standard? | Yes | No | Unclear |
| 3. Did all patients receive the same reference standard? | Yes | No | Unclear |
| 4. Were all patients included in the analysis? Most of the time, this answer will be Yes. There should be results for all patients/specimens including indeterminate or unable to perform test. | Yes | No | Unclear (Do not use this answer for this question) |
| Could the patient flow have introduced bias? | High (Do not use High as answer for this question) | Low | Unclear |
| Score Low Risk of Bias for 4. Yes  Score Unclear Risk of Bias for 4. No |  |  |  |

Low risk of bias studies will require low risk of bias in Domains 1 (patient selection/representative population and 2 (index test blinding)

Domain 3 concerns the reference standard and is pre-specified as low risk of bias as inclusion criterion

Domain4 concerns flow and timing of test and is expected to be low risk of bias for the majority of studies

**Appendix S4: references for included and excluded studies**

**Included studies**

1. Affolabi, D., et al., *Evaluation of direct detection of Mycobacterium tuberculosis rifampin resistance by a nitrate reductase assay applied to sputum samples in Cotonou, Benin.* J Clin Microbiol, 2007. **45**(7): p. 2123-5.

2. Affolabi, D., et al., *Rapid and inexpensive detection of multidrug-resistant Mycobacterium tuberculosis with the nitrate reductase assay using liquid medium and direct application to sputum samples.* J Clin Microbiol, 2008. **46**(10): p. 3243-5.

3. Affolabi, D., et al., *Rapid detection of multidrug-resistant Mycobacterium tuberculosis in Cotonou (Benin) using two low-cost colorimetric methods: resazurin and nitrate reductase assays.* J Med Microbiol, 2008. **57**(Pt 8): p. 1024-7.

4. Ahmad, S., N.M. Al-Mutairi, and E. Mokaddas, *Comparison of performance of two DNA line probe assays for rapid detection of multidrug-resistant isolates of Mycobacterium tuberculosis.* Indian J Exp Biol, 2009. **47**(6): p. 454-62.

5. Albert, H., et al., *Rapid screening of MDR-TB using molecular Line Probe Assay is feasible in Uganda.* BMC Infect Dis, 2010. **10**: p. 41.

6. Anek-Vorapong, R., et al., *Validation of the GenoType MTBDRplus assay for detection of MDR-TB in a public health laboratory in Thailand.* BMC Infect Dis, 2010. **10**: p. 123.

7. Ani, A.E., et al., *Drug susceptibility test of Mycobacterium tuberculosis by nitrate reductase assay.* J Infect Dev Ctries, 2009. **3**(1): p. 16-9.

8. Asencios, L., et al., *Programmatic implementation of rapid DST for Mycobacterium tuberculosis in Peru.* Int J Tuberc Lung Dis, 2008. **12**(7): p. 743-9.

9. Banfi, E., G. Scialino, and C. Monti-Bragadin, *Development of a microdilution method to evaluate Mycobacterium tuberculosis drug susceptibility.* J Antimicrob Chemother, 2003. **52**(5): p. 796-800.

10. Barnard, M., et al., *Rapid molecular screening for multidrug-resistant tuberculosis in a high-volume public health laboratory in South Africa.* Am J Respir Crit Care Med, 2008. **177**(7): p. 787-92.

11. Bwanga, F., et al., *Direct nitrate reductase assay versus microscopic observation drug susceptibility test for rapid detection of MDR-TB in Uganda.* PLoS One, 2011. **6**(5): p. e19565.

12. Chauca, J.A., J.C. Palomino, and H. Guerra, *Evaluation of the accuracy of the microplate Alamar Blue assay for rapid detection of MDR-TB in Peru.* Int J Tuberc Lung Dis, 2007. **11**(7): p. 820-2.

13. Cirillo, D.M., et al., *Direct rapid diagnosis of rifampicin-resistant M. tuberculosis infection in clinical samples by line probe assay (INNO LiPA Rif-TB).* New Microbiol, 2004. **27**(3): p. 221-7.

14. Coban, A.Y., et al., *Drug susceptibility testing of Mycobacterium tuberculosis with nitrate reductase assay.* Int J Antimicrob Agents, 2004. **24**(3): p. 304-6.

15. de la Iglesia, A.I., E.J. Stella, and H.R. Morbidoni, *Comparison of the performances of two in-house rapid methods for antitubercular drug susceptibility testing.* Antimicrob Agents Chemother, 2009. **53**(2): p. 808-10.

16. Duo, L., et al., *Molecular profile of drug resistance in tuberculous meningitis from southwest china.* Clin Infect Dis, 2011. **53**(11): p. 1067-73.

17. Ejigu, G.S., et al., *Microscopic-observation drug susceptibility assay provides rapid and reliable identification of MDR-TB.* Int J Tuberc Lung Dis, 2008. **12**(3): p. 332-7.

18. Evans, J., et al., *Rapid genotypic assays to identify drug-resistant Mycobacterium tuberculosis in South Africa.* J Antimicrob Chemother, 2009. **63**(1): p. 11-6.

19. Franzblau, S.G., et al., *Rapid, low-technology MIC determination with clinical Mycobacterium tuberculosis isolates by using the microplate Alamar Blue assay.* J Clin Microbiol, 1998. **36**(2): p. 362-6.

20. Giacomazzi, C.G., et al., *Rapid diagnosis of tuberculosis and multidrug resistance with the microscopic observation drug susceptibility assay in Ecuador.* Int J Tuberc Lung Dis, 2010. **14**(6): p. 786-8.

21. Gitti, Z., et al., *GenoType(R) MTBDRplus compared with conventional drug-susceptibility testing of Mycobacterium tuberculosis in a low-resistance locale.* Future Microbiol, 2011. **6**(3): p. 357-62.

22. Gupta, A. and S. Anupurba, *Direct drug susceptibility testing of Mycobacterium tuberculosis against primary anti-TB drugs in northern India.* J Infect Dev Ctries, 2010. **4**(11): p. 695-703.

23. Gupta, A., et al., *Evaluation of the performance of nitrate reductase assay for rapid drug-susceptibility testing of mycobacterium tuberculosis in north India.* J Health Popul Nutr, 2011. **29**(1): p. 20-5.

24. Gupta, M., N.P. Singh, and I.R. Kaur, *Evaluation of nitrate reductase assay for direct detection of drug resistance in Mycobacterium tuberculosis: rapid and inexpensive method for low-resource settings.* Indian J Med Microbiol, 2010. **28**(4): p. 363-5.

25. Hillemann, D., S. Rusch-Gerdes, and E. Richter, *Application of the Genotype MTBDR assay directly on sputum specimens.* Int J Tuberc Lung Dis, 2006. **10**(9): p. 1057-9.

26. Huang, W.L., et al., *Performance assessment of the GenoType MTBDRplus test and DNA sequencing in detection of multidrug-resistant Mycobacterium tuberculosis.* Journal of Clinical Microbiology, 2009. **47 (8)**: p. 2520-2524.

27. Huyen, M.N., et al., *Validation of the GenoType MTBDRplus assay for diagnosis of multidrug resistant tuberculosis in South Vietnam.* BMC Infect Dis, 2010. **10**: p. 149.

28. Lacoma, A., et al., *GenoType MTBDRplus assay for molecular detection of rifampin and isoniazid resistance in Mycobacterium tuberculosis strains and clinical samples.* J Clin Microbiol, 2008. **46**(11): p. 3660-7.

29. Limaye, K., et al., *Utility of Microscopic Observation of Drug Susceptibility (MODS) assay for Mycobacterium tuberculosis in resource constrained settings.* Indian J Tuberc, 2010. **57**(4): p. 207-12.

30. Luna-Herrera, J., et al., *Use of receiver operating characteristic curves to assess the performance of a microdilution assay for determination of drug susceptibility of clinical isolates of Mycobacterium tuberculosis.* Eur J Clin Microbiol Infect Dis, 2003. **22**(1): p. 21-7.

31. Makinen, J., et al., *Comparison of two commercially available DNA line probe assays for detection of multidrug-resistant Mycobacterium tuberculosis.* J Clin Microbiol, 2006. **44**(2): p. 350-2.

32. Mello, F.C., et al., *Clinical evaluation of the microscopic observation drug susceptibility assay for detection of Mycobacterium tuberculosis resistance to isoniazid or rifampin.* J Clin Microbiol, 2007. **45**(10): p. 3387-9.

33. Mendoza, R., et al., *[Susceptibility of M. tuberculosis to antituberculosis drugs as determined by two methods, in Sucre state, Venezuela].* Invest Clin, 2010. **51**(4): p. 445-55.

34. Mengatto, L., Y. Chiani, and M.S. Imaz, *Evaluation of rapid alternative methods for drug susceptibility testing in clinical isolates of Mycobacterium tuberculosis.* Mem Inst Oswaldo Cruz, 2006. **101**(5): p. 535-42.

35. Miyata, M., et al., *Drug resistance in Mycobacterium tuberculosis clinical isolates from Brazil: Phenotypic and genotypic methods.* Biomedicine and Pharmacotherapy, 2011. **65 (6)**: p. 456-459.

36. Moore, D.A., et al., *Microscopic-observation drug-susceptibility assay for the diagnosis of TB.* N Engl J Med, 2006. **355**(15): p. 1539-50.

37. Morcillo, N., B. Imperiale, and B. Di Giulio, *Evaluation of MGIT 960 and the colorimetric-based method for tuberculosis drug susceptibility testing.* Int J Tuberc Lung Dis, 2010. **14**(9): p. 1169-75.

38. Musa, H.R., et al., *Drug susceptibility testing of Mycobacterium tuberculosis by a nitrate reductase assay applied directly on microscopy-positive sputum samples.* J Clin Microbiol, 2005. **43**(7): p. 3159-61.

39. Nateche, F., et al., *Application of the resazurin microtitre assay for detection of multidrug resistance in Mycobacterium tuberculosis in Algiers.* J Med Microbiol, 2006. **55**(Pt 7): p. 857-60.

40. Ogwang, S., et al., *Comparison of rapid tests for detection of rifampicin-resistant Mycobacterium tuberculosis in Kampala, Uganda.* BMC Infect Dis, 2009. **9**: p. 139.

41. Palomino, J.C., et al., *Resazurin microtiter assay plate: simple and inexpensive method for detection of drug resistance in Mycobacterium tuberculosis.* Antimicrob Agents Chemother, 2002. **46**(8): p. 2720-2.

42. Palomino, J.C. and F. Portaels, *Simple procedure for drug susceptibility testing of Mycobacterium tuberculosis using a commercial colorimetic assay.* Eur J Clin Microbiol Infect Dis, 1999. **18**(5): p. 380-3.

43. Park, W.G., et al., *Performance of the microscopic observation drug susceptibility assay in drug susceptibility testing for Mycobacterium tuberculosis.* J Clin Microbiol, 2002. **40**(12): p. 4750-2.

44. Pontino, M.V., et al., *[Evaluation of a colorimetric micromethod for determining the minimal inhibitory concentration of antibiotics against Mycobacterium tuberculosis].* Rev Argent Microbiol, 2006. **38**(3): p. 145-51.

45. Raut, U., et al., *Evaluation of rapid MTT tube method for detection of drug susceptibility of Mycobacterium tuberculosis to rifampicin and isoniazid.* Indian J Med Microbiol, 2008. **26**(3): p. 222-7.

46. Reis, R.S., et al., *Comparison of flow cytometric and Alamar Blue tests with the proportional method for testing susceptibility of Mycobacterium tuberculosis to rifampin and isoniazid.* J Clin Microbiol, 2004. **42**(5): p. 2247-8.

47. Rigouts, L., et al., *Evaluation of the Genotype(R) MTBDRplus assay as a tool for drug resistance surveys.* Int J Tuberc Lung Dis, 2011. **15**(7): p. 959-65.

48. Rosales, S., et al., *Field assessment of the direct nitrate reductase assay for rapid detection of multidrug-resistant tuberculosis in Honduras.* Int J Tuberc Lung Dis, 2011. **15**(9): p. 1206-10, i.

49. Sam, I.C., et al., *Mycobacterium tuberculosis and rifampin resistance, United Kingdom.* Emerg Infect Dis, 2006. **12**(5): p. 752-9.

50. Scott, L.E., et al., *Comparison of Xpert MTB/RIF with other nucleic acid technologies for diagnosing pulmonary tuberculosis in a high HIV prevalence setting: a prospective study.* PLoS Med, 2011. **8**(7): p. e1001061.

51. Sethi, S., et al., *Drug susceptibility of Mycobacterium tuberculosis to primary antitubercular drugs by nitrate reductase assay.* Indian J Med Res, 2004. **120**(5): p. 468-71.

52. Shah, N.S., et al., *Rapid diagnosis of tuberculosis and multidrug resistance by the microscopic-observation drug-susceptibility assay.* Am J Respir Crit Care Med, 2011. **183**(10): p. 1427-33.

53. Shiferaw, G., et al., *Evaluation of microscopic observation drug susceptibility assay for detection of multidrug-resistant Mycobacterium tuberculosis.* J Clin Microbiol, 2007. **45**(4): p. 1093-7.

54. Shikama Mde, L., et al., *Rapid detection of resistant tuberculosis by nitrate reductase assay performed in three settings in Brazil.* J Antimicrob Chemother, 2009. **64**(4): p. 794-6.

55. Shikama, M.L., et al., *Multicentre study of nitrate reductase assay for rapid detection of rifampicin-resistant M. tuberculosis.* Int J Tuberc Lung Dis, 2009. **13**(3): p. 377-80.

56. Skenders, G., et al., *Implementation of the INNO-LiPA Rif.TB line-probe assay in rapid detection of multidrug-resistant tuberculosis in Latvia.* International Journal of Tuberculosis and Lung Disease, 2011. **15 (11)**: p. 1546-1552.

57. Solis, L.A., et al., *Validation of a rapid method for detection of M. tuberculosis resistance to isoniazid and rifampin in Lima, Peru.* Int J Tuberc Lung Dis, 2005. **9**(7): p. 760-4.

58. Somoskovi, A., et al., *Use of smear-positive samples to assess the PCR-based genotype MTBDR assay for rapid, direct detection of the Mycobacterium tuberculosis complex as well as its resistance to isoniazid and rifampin.* J Clin Microbiol, 2006. **44**(12): p. 4459-63.

59. Visalakshi, P., et al., *Evaluation of direct method of drug susceptibility testing of Mycobacterium tuberculosis to rifampicin and isoniazid by nitrate reductase assay in a national reference laboratory.* Diagn Microbiol Infect Dis, 2010. **66**(2): p. 148-52.

60. Mironova, S., et al., *Performance of the GenoType((R)) MTBDRPlus assay in routine settings: a multicenter study.* Eur J Clin Microbiol Infect Dis, 2011.

**Excluded studies and reasons for exclusion**

1. Akpaka, P.E., et al., *Evaluation of methods for rapid detection of resistance to isoniazid and rifampin in Mycobacterium tuberculosis isolates collected in the Caribbean.* J Clin Microbiol, 2008. **46**(10): p. 3426-8.

Insufficient data

2. Al-Mutairi, N., S. Ahmad, and E. Mokaddas, *Performance of the genotype MTBDR assay for molecular detection of multidrug-resistant strains of Mycobacterium tuberculosis.* Ann Saudi Med, 2008. **28**(3): p. 203-6.

Case Control study design

3. Al-Mutairi, N.M., S. Ahmad, and E. Mokaddas, *Performance comparison of four methods for detecting multidrug-resistant Mycobacterium tuberculosis strains.* Int J Tuberc Lung Dis, 2011. **15**(1): p. 110-5.

Case Control study design

4. Amor, Y.B., M. Fraden, and J. Ruxin, *Reversing the tide of tuberculosis in India: Complementing microscopy with line probe assays.* Global Public Health, 2008. **3 (4)**: p. 399-416.

Review or editorial

5. Ando, H., et al., *Pyrazinamide resistance in multidrug-resistant Mycobacterium tuberculosis isolates in Japan.* Clin Microbiol Infect, 2010. **16**(8): p. 1164-8.

No testing for rifampicin resistance

6. Angeby, K.A., L. Klintz, and S.E. Hoffner, *Rapid and inexpensive drug susceptibility testing of Mycobacterium tuberculosis with a nitrate reductase assay.* J Clin Microbiol, 2002. **40**(2): p. 553-5.

Case Control study design

7. Arias, M., et al., *Clinical evaluation of the microscopic-observation drug-susceptibility assay for detection of tuberculosis.* Clin Infect Dis, 2007. **44**(5): p. 674-80.

No testing for rifampicin resistance

8. Balabanova, Y., et al., *An integrated approach to rapid diagnosis of tuberculosis and multidrug resistance using liquid culture and molecular methods in Russia.* PLoS One, 2009. **4**(9): p. e7129.

Insufficient data

9. Bang, D., A. Bengard Andersen, and V.O. Thomsen, *Rapid genotypic detection of rifampin- and isoniazid-resistant Mycobacterium tuberculosis directly in clinical specimens.* J Clin Microbiol, 2006. **44**(7): p. 2605-8.

Case Control study design

10. Bazira, J., et al., *Use of the Genotype MTBDRplus assay to assess drug resistance of Mycobacterium tuberculosis isolates from patients in rural Uganda.* BMC Clinical Pathology, 2010. **10**(5).

Insufficient data

11. Bicmen, C., et al., *Molecular identification and characterization of rifampicin-resistant Mycobacterium tuberculosis isolates by line probe assay: an approach for rapid diagnosis of multidrug-resistant tuberculosis.* Lett Appl Microbiol, 2008. **47**(3): p. 214-20.

Case Control study design

12. Brady, M.F., et al., *The MODS method for diagnosis of tuberculosis and multidrug resistant tuberculosis.* J Vis Exp, 2008(17).

Review or editorial

13. Brossier, F., et al., *Detection by GenoType MTBDRsl test of complex mechanisms of resistance to second-line drugs and ethambutol in multidrug-resistant Mycobacterium tuberculosis complex isolates.* J Clin Microbiol, 2010. **48**(5): p. 1683-9.

Insufficient data

14. Brossier, F., et al., *Performance of MTBDR plus for detecting high/low levels of Mycobacterium tuberculosis resistance to isoniazid.* Int J Tuberc Lung Dis, 2009. **13**(2): p. 260-5.

Case Control study design

15. Brossier, F., et al., *Performance of the genotype MTBDR line probe assay for detection of resistance to rifampin and isoniazid in strains of Mycobacterium tuberculosis with low- and high-level resistance.* J Clin Microbiol, 2006. **44**(10): p. 3659-64.

Insufficient data

16. Bwanga, F., et al., *Evaluation of seven tests for the rapid detection of multidrug-resistant tuberculosis in Uganda.* Int J Tuberc Lung Dis, 2010. **14**(7): p. 890-5.

Case Control study design

17. Campanerut, P.A., et al., *Rapid detection of resistance to pyrazinamide in Mycobacterium tuberculosis using the resazurin microtitre assay.* J Antimicrob Chemother, 2011. **66**(5): p. 1044-6.

No testing for rifampicin resistance

18. Carvalho, W.D.S., et al., *Diagnostic of rifampicin-resistance of Mycobacterium tuberculosis by polymerase chain reaction. [Portuguese].* Revista Brasileira de Ciencias Farmaceuticas/Brazilian Journal of Pharmaceutical Sciences, 2007. **43 (1)**: p. 31-38.

Excluded language

19. Causse, M., et al., *Evaluation of new GenoType MTBDRplus for detection of resistance in cultures and direct specimens of Mycobacterium tuberculosis.* Int J Tuberc Lung Dis, 2008. **12**(12): p. 1456-60.

Case Control study design

20. Caviedes, L., et al., *Rapid, efficient detection and drug susceptibility testing of Mycobacterium tuberculosis in sputum by microscopic observation of broth cultures. The Tuberculosis Working Group in Peru.* J Clin Microbiol, 2000. **38**(3): p. 1203-8.

Lack of an accepted reference standard

21. Caviedes, L. and D.A. Moore, *Introducing MODS: a low-cost, low-tech tool for high-performance detection of tuberculosis and multidrug resistant tuberculosis.* Indian J Med Microbiol, 2007. **25**(2): p. 87-8.

Review or editorial

22. Cavusoglu, C., D. Gursel, and H.B. Aktoprak, *Evaluation of the genotype MTBDRplus assay for the diagnosis of tuberculosis and rapid detection of rifampin and isoniazid resistance in clinical specimens. [Turkish].* Turkish Journal of Medical Sciences, 2011. **41 (3)**: p. 419-425.

Excluded language

23. Cavusoglu, C., et al., *Evaluation of the Genotype MTBDR assay for rapid detection of rifampin and isoniazid resistance in Mycobacterium tuberculosis isolates.* J Clin Microbiol, 2006. **44**(7): p. 2338-42.

Case Control study design

24. Caws, M., et al., *Evaluation of the MODS culture technique for the diagnosis of tuberculous meningitis.* PLoS One, 2007. **2**(11): p. e1173.

No testing for rifampicin resistance

25. Chedore, P., et al., *Potential for erroneous results indicating resistance when using the Bactec MGIT 960 system for testing susceptibility of Mycobacterium tuberculosis to pyrazinamide.* J Clin Microbiol, 2010. **48**(1): p. 300-1.

No testing for rifampicin resistance

26. Coban, A.Y., et al., *A rapid detection of multidrug-resistant Mycobacterium tuberculosis by a nitrate reductase assay on blood agar.* Mem Inst Oswaldo Cruz, 2011. **106**(3): p. 378-80.

Lack of included index test

27. Coban, A.Y., et al., *Rapid susceptibility test for Mycobacterium tuberculosis to isoniazid and rifampin with resazurin method in screw-cap tubes.* J Chemother, 2006. **18**(2): p. 140-3.

Case Control study design

28. Comina, G., et al., *Development of an automated MODS plate reader to detect early growth of Mycobacterium tuberculosis.* J Microsc, 2011. **242**(3): p. 325-30.

No testing for rifampicin resistance

29. Coronel, J., et al., *MODS accreditation process for regional reference laboratories in Peru: validation by GenoType(R) MTBDRplus.* Int J Tuberc Lung Dis, 2010. **14**(11): p. 1475-80.

Lack of an accepted reference standard

30. Cuevas-Cordoba, B. and R. Zenteno-Cuevas, *Drug resistant tuberculosis: Molecular mechanisms and diagnostic methods. [Spanish].* Enfermedades Infecciosas y Microbiologia Clinica, 2010. **28 (9)**: p. 621-628.

Review or editorial

31. da Silva, P.A., et al., *Comparison of redox and D29 phage methods for detection of isoniazid and rifampicin resistance in Mycobacterium tuberculosis.* Clin Microbiol Infect, 2006. **12**(3): p. 293-6.

Case Control study design

32. De Kantor, I.N., *Validation of a rapid method for detection of M. tuberculosis resistance to INH and RMP in Lima, Peru [1].* International Journal of Tuberculosis and Lung Disease, 2006. **10 (6)**: p. 707.

Insufficient data

33. Devasia, R.A., et al., *Fluoroquinolone resistance in Mycobacterium tuberculosis: an assessment of MGIT 960, MODS and nitrate reductase assay and fluoroquinolone cross-resistance.* J Antimicrob Chemother, 2009. **63**(6): p. 1173-8.

No testing for rifampicin resistance

34. El-Sayed Zaki, M. and T. Goda, *Rapid phenotypic assay of antimycobacterial susceptibility pattern by direct mycobacteria growth indicator tube and phage amplified biological assay compared to BACTEC 460 TB.* Tuberculosis (Edinb), 2007. **87**(2): p. 102-8.

Lack of included index test

35. Fabre, M., et al., *[Performances of the assay MTBDRplus((R)) in the surveillance of rifampicin resistance in Mycobacterium tuberculosis].* Pathol Biol (Paris), 2011. **59**(2): p. 94-6.

Lack of an accepted reference standard

36. Farnia, P., et al., *Colorimetric detection of multidrug-resistant or extensively drug-resistant tuberculosis by use of malachite green indicator dye.* J Clin Microbiol, 2008. **46**(2): p. 796-9.

Lack of included index test

37. Fegou, E., et al., *Comparison of the manual Mycobacteria Growth Indicator tube and the Etest with the method of proportion for susceptibility testing of Mycobacterium tuberculosis.* Chemotherapy, 2006. **52**(4): p. 174-7.

Lack of included index test

38. Ferrari Mde, L., et al., *Susceptibility of Mycobacterium tuberculosis to first-line antimycobacterial agents in a Brazilian hospital: assessing the utility of the tetrazolium (MTT) microplate assay.* Mem Inst Oswaldo Cruz, 2010. **105**(5): p. 661-4.

Lack of an acceptable reference standard

39. Foongladda, S., et al., *Rapid and simple MTT method for rifampicin and isoniazid susceptibility testing of Mycobacterium tuberculosis.* Int J Tuberc Lung Dis, 2002. **6**(12): p. 1118-22.

Case Control study design

40. Gui, X.H., et al., *[Evaluation of the GenoType MTBDRplus assay for rifampin and isoniazid susceptibility testing of Mycobacterium tuberculosis].* Zhonghua Jie He He Hu Xi Za Zhi, 2010. **33**(1): p. 43-5.

Excluded language

41. Ha, D.T., et al., *Diagnosis of pulmonary tuberculosis in HIV-positive patients by microscopic observation drug susceptibility assay.* J Clin Microbiol, 2010. **48**(12): p. 4573-9.

Lack of an accepted reference standard

42. Ha, D.T., et al., *Microscopic observation drug susceptibility assay (MODS) for early diagnosis of tuberculosis in children.* PLoS One, 2009. **4**(12): p. e8341.

No testing for rifampicin resistance

43. Hasan, R. and S. Irfan, *MODS assay for the diagnosis of TB.* N Engl J Med, 2007. **356**(2): p. 188; author reply 189.

Insufficient data

44. Hauck, Y., et al., *Comparison of two commercial assays for the characterization of rpoB mutations in Mycobacterium tuberculosis and description of new mutations conferring weak resistance to rifampicin.* J Antimicrob Chemother, 2009. **64**(2): p. 259-62.

Case Control study design

45. Hillemann, D., S. Rusch-Gerdes, and E. Richter, *Evaluation of the GenoType MTBDRplus assay for rifampin and isoniazid susceptibility testing of Mycobacterium tuberculosis strains and clinical specimens.* J Clin Microbiol, 2007. **45**(8): p. 2635-40.

Case Control study design

46. Hillemann, D., et al., *Use of the genotype MTBDR assay for rapid detection of rifampin and isoniazid resistance in Mycobacterium tuberculosis complex isolates.* J Clin Microbiol, 2005. **43**(8): p. 3699-703.

Case Control study design

47. Ho, J.E., *Gastric aspirate culture using the microscopic observation drug susceptibility (MODS) technique is superior to other methods for bacteriological confirmation of tuberculosis in children.* Thorax, 2011. **66 (7)**: p. 608.

No testing for rifampicin resistance

48. Hoek, K.G., et al., *Detecting drug-resistant tuberculosis: the importance of rapid testing.* Mol Diagn Ther, 2011. **15**(4): p. 189-94.

Review or editorial

49. Huang, W.L., et al., *Performance assessment of the GenoType MTBDRsl test and DNA sequencing for detection of second-line and ethambutol drug resistance among patients infected with multidrug-resistant Mycobacterium tuberculosis.* J Clin Microbiol, 2011. **49**(7): p. 2502-8.

No testing for rifampicin resistance

50. Jin, W.G., et al., *[Detection on drug resistance of Mycobacterium tuberculosis by microscopic observation drug susceptibility].* Zhonghua Yu Fang Yi Xue Za Zhi, 2009. **43**(1): p. 24-7.

Excluded language

51. John, O., *Microscopic-observation drug-susceptibility assay was more sensitive than standard tests for diagnosing tuberculosis.* ACP J Club, 2007. **146**(2): p. 45.

Review or editorial

52. Johnson, R., et al., *Drug susceptibility testing using molecular techniques can enhance tuberculosis diagnosis.* J Infect Dev Ctries, 2008. **2**(1): p. 40-5.

Lack of included index test

53. Jou, R., et al., *Proficiency of drug susceptibility testing for Mycobacterium tuberculosis in Taiwan.* Int J Tuberc Lung Dis, 2009. **13**(9): p. 1142-7.

Case Control study design

54. Kumar, M., et al., *Rapid, inexpensive MIC determination of Mycobacterium tuberculosis isolates by using microplate nitrate reductase assay.* Diagn Microbiol Infect Dis, 2005. **53**(2): p. 121-4.

Case Control study design

55. Kumar, M., et al., *Microplate nitrate reductase assay versus Alamar Blue assay for MIC determination of Mycobacterium tuberculosis.* Int J Tuberc Lung Dis, 2005. **9**(8): p. 939-41.

Lack of an accepted reference standard

56. Lee, S., et al., *Evaluation of a modified antimycobacterial susceptibility test using Middlebrook 7H10 agar containing 2,3-diphenyl-5-thienyl-(2)-tetrazolium chloride.* J Microbiol Methods, 2006. **66**(3): p. 548-51.

Case Control study design

57. Lemus, D., et al., *Rapid alternative methods for detection of rifampicin resistance in Mycobacterium tuberculosis.* J Antimicrob Chemother, 2004. **54**(1): p. 130-3.

Case Control study design

58. Lemus, D., et al., *Nitrate reductase assay for detection of drug resistance in Mycobacterium tuberculosis: simple and inexpensive method for low-resource laboratories.* J Med Microbiol, 2006. **55**(Pt 7): p. 861-3.

Case Control study design

59. Leonard, B., et al., *Inter- and intra-assay reproducibility of microplate Alamar blue assay results for isoniazid, rifampicin, ethambutol, streptomycin, ciprofloxacin, and capreomycin drug susceptibility testing of Mycobacterium tuberculosis.* J Clin Microbiol, 2008. **46**(10): p. 3526-9.

Insufficient data

60. Macedo, R., A. Amorim, and E. Pereira, *Multidrug-resistant tuberculosis: rapid molecular detection with MTBDRplus assay in clinical samples.* Rev Port Pneumol, 2009. **15**(3): p. 353-65.

Lack of an accepted reference standard

61. Martin, A., et al., *Multicenter evaluation of the nitrate reductase assay for drug resistance detection of Mycobacterium tuberculosis.* J Microbiol Methods, 2005. **63**(2): p. 145-50.

Case Control study design

62. Martin, A., et al., *Multicenter study of MTT and resazurin assays for testing susceptibility to first-line anti-tuberculosis drugs.* Int J Tuberc Lung Dis, 2005. **9**(8): p. 901-6.

Case Control study design

63. Martin, A., et al., *Multicentre laboratory validation of the colorimetric redox indicator (CRI) assay for the rapid detection of extensively drug-resistant (XDR) Mycobacterium tuberculosis.* J Antimicrob Chemother, 2011. **66**(4): p. 827-33.

Case Control study design

64. Mashta, A., et al., *Diagnosis of Tuberculosis : the experience at a specialized diagnostic laboratory.* J Negat Results Biomed, 2011. **10**(1): p. 16.

No testing for rifampicin resistance

65. Mendoza, A., et al., *Reliability of the MODS assay decentralisation process in three health regions in Peru.* Int J Tuberc Lung Dis, 2011. **15**(2): p. 217-22, i.

Insufficient data

66. Michael, J.S., et al., *Diagnostic accuracy of the microscopic observation drug susceptibility assay: a pilot study from India.* Int J Tuberc Lung Dis, 2010. **14**(4): p. 482-8.

Insufficient data

67. Minime-Lingoupou, F., et al., *Rapid identification of multidrug-resistant tuberculosis isolates in treatment failure or relapse patients in Bangui, Central African Republic.* Int J Tuberc Lung Dis, 2010. **14**(6): p. 782-5.

Insufficient data

68. Miotto, P., et al., *Genotype MTBDRplus: a further step toward rapid identification of drug-resistant Mycobacterium tuberculosis.* J Clin Microbiol, 2008. **46**(1): p. 393-4.

Lack of an accepted reference standard

69. Miotto, P., et al., *Use of genotype MTBDR assay for molecular detection of rifampin and isoniazid resistance in Mycobacterium tuberculosis clinical strains isolated in Italy.* J Clin Microbiol, 2006. **44**(7): p. 2485-91.

Case Control study design

70. Miotto, P., et al., *Molecular detection of rifampin and isoniazid resistance to guide chronic TB patient management in Burkina Faso.* BMC Infect Dis, 2009. **9**: p. 142.

Abstract

71. Mishra, B., S. Muralidharan, and H. Srinivasa, *Direct drug susceptibility testing of Mycobacterium tuberculosis to primary anti-tubercular drugs by nitrate reductase assay.* Indian J Pathol Microbiol, 2009. **52**(3): p. 343-4.

Insufficient data

72. Mohammadzadeh, A., et al., *Rapid and low-cost colorimetric method using 2,3,5-triphenyltetrazolium chloride for detection of multidrug-resistant Mycobacterium tuberculosis.* Journal of Medical Microbiology, 2006. **55 (12)**: p. 1657-1659.

Case Control study design

73. Mokaddas, M.A., N. Al Mutairi, and S. Ahmed, *Comparison of INNO-LiPA Rif. TB, genotype MTBDRplus, PCR-RFLP and DNA sequencing for detecting multidrug-resistant Mycobacterium tuberculosis isolates.* Clinical Microbiology and Infection, 2010. **Conference: 20th ECCMID Vienna Austria. Conference Start: 20100410 Conference End: 20100413. Conference Publication: (var.pagings). 16**: p. S613.

Abstract

74. Montoro, E., et al., *Comparative evaluation of the nitrate reduction assay, the MTT test, and the resazurin microtitre assay for drug susceptibility testing of clinical isolates of Mycobacterium tuberculosis.* J Antimicrob Chemother, 2005. **55**(4): p. 500-5.

Case Control study design

75. Moore, D.A., et al., *Infrequent MODS TB culture cross-contamination in a high-burden resource-poor setting.* Diagn Microbiol Infect Dis, 2006. **56**(1): p. 35-43.

Insufficient data

76. Moore, D.A., et al., *Microscopic observation drug susceptibility assay, a rapid, reliable diagnostic test for multidrug-resistant tuberculosis suitable for use in resource-poor settings.* J Clin Microbiol, 2004. **42**(10): p. 4432-7.

Lack of an accepted reference standard

77. Negi, S.S., et al., *Molecular characterization of mutation associated with rifampicin and isoniazid resistance in Mycobacterium tuberculosis isolates.* Indian J Exp Biol, 2006. **44**(7): p. 547-53.

Case Control study design

78. Neonakis, I.K., et al., *Evaluation of GenoType mycobacteria direct assay in comparison with gen-probe Mycobacterium tuberculosis amplified direct test and GenoType MTBDRplus for direct detection of Mycobacterium tuberculosis complex in clinical samples.* Journal of Clinical Microbiology, 2009. **47 (8)**: p. 2601-2603.

No testing for rifampicin resistance

79. Nic Fhogartaigh, C.J., et al., *Physician-initiated courtesy MODS testing for TB and MDR-TB diagnosis and patient management.* Int J Tuberc Lung Dis, 2008. **12**(5): p. 555-60.

Insufficient data

80. Nikolayevskyy, V., et al., *Performance of the Genotype MTBDRPlus assay in the diagnosis of tuberculosis and drug resistance in Samara, Russian Federation.* BMC Clin Pathol, 2009. **9**: p. 2.

Insufficient data

81. Oberhelman, R.A., et al., *Improved recovery of Mycobacterium tuberculosis from children using the microscopic observation drug susceptibility method.* Pediatrics, 2006. **118**(1): p. e100-6.

No testing for rifampicin resistance

82. Oberhelman, R.A., et al., *Diagnostic approaches for paediatric tuberculosis by use of different specimen types, culture methods, and PCR: a prospective case-control study.* Lancet Infect Dis, 2010. **10**(9): p. 612-20.

No testing for rifampicin resistance

83. Ozkutuk, N., et al., *Characterization of rpoB mutations by line probe assay in rifampicin-resistant mycobacterium tuberculosis clinical isolates from the aegean region in Turkey.* Jpn J Infect Dis, 2007. **60**(4): p. 211-3.

Case Control study design

84. Paluch-Oles, J., M. Koziol-Montewka, and A. Magrys, *Mutations in the rpoB gene of rifampin-resistant Mycobacterium tuberculosis isolates from Eastern Poland.* New Microbiol, 2009. **32**(2): p. 147-52.

Lack of an accepted reference standard

85. Panaiotov, S., *Crystalline nitrate reductase reagent for drug susceptibility testing of Mycobacterium tuberculosis.* Int J Antimicrob Agents, 2009. **34**(2): p. 191-2.

Insufficient data

86. Panaiotov, S. and T. Kantardjiev, *Nitrate reductase assay for drug susceptibility testing of Mycobacterium tuberculosis.* J Clin Microbiol, 2002. **40**(10): p. 3881; author reply 3881-2.

Insufficient data

87. Poojary, A., et al., *Rapid antibiotic susceptibility testing of Mycobacterium tuberculosis: its utility in resource poor settings.* Indian J Med Microbiol, 2006. **24**(4): p. 268-72.

Lack of included index test

88. Quezada, C.M., et al., *Implementation validation performed in Rwanda to determine whether the INNO-LiPA Rif.TB line probe assay can be used for detection of multidrug-resistant Mycobacterium tuberculosis in low-resource countries.* J Clin Microbiol, 2007. **45**(9): p. 3111-4.

Case Control study design

89. Reddy, K.P., et al., *Microscopic observation drug susceptibility assay for tuberculosis screening before isoniazid preventive therapy in HIV-infected persons.* Clin Infect Dis, 2010. **50**(7): p. 988-96.

No testing for rifampicin resistance

90. Reddy, S., T. Brown, and F. Drobniewski, *Detection of Mycobacterium tuberculosis from paraffin-embedded tissues by INNO-LiPA Rif.TB assay: retrospective analyses of Health Protection Agency National Mycobacterium Reference Laboratory data.* J Med Microbiol, 2010. **59**(Pt 5): p. 563-6.

No resistance to rifampicin identified

91. Rivoire, N., et al., *Evaluation of the resazurin assay for the detection of multidrug-resistant Mycobacterium tuberculosis in Madagascar.* International Journal of Tuberculosis and Lung Disease, 2007. **11 (6)**: p. 683-688.

Case Control study design

92. Rosales, S., et al., *Evaluation of the nitrate reductase assay for rapid detection of extensively drug-resistant tuberculosis.* Int J Tuberc Lung Dis, 2009. **13**(12): p. 1542-9.

Case Control study design

93. Seagar, A.L., et al., *Evaluation of the GenoType Mycobacteria Direct assay for the simultaneous detection of the Mycobacterium tuberculosis complex and four atypical mycobacterial species in smear-positive respiratory specimens.* J Med Microbiol, 2008. **57**(Pt 5): p. 605-11.

Abstract

94. Sekiguchi, J., et al., *Development and evaluation of a line probe assay for rapid identification of pncA mutations in pyrazinamide-resistant mycobacterium tuberculosis strains.* J Clin Microbiol, 2007. **45**(9): p. 2802-7.

No testing for rifampicin resistance

95. Shah, N.S., et al., *Validation of the line-probe assay for rapid detection of rifampicin-resistant Mycobacterium tuberculosis in Vietnam.* Int J Tuberc Lung Dis, 2009. **13**(2): p. 247-52.

Case Control study design

96. Singh, P., et al., *Comparative evaluation of Lowenstein-Jensen proportion method, BacT/ALERT 3D system, and enzymatic pyrazinamidase assay for pyrazinamide susceptibility testing of Mycobacterium tuberculosis.* J Clin Microbiol, 2007. **45**(1): p. 76-80.

No testing for rifampicin resistance

97. Soudani, A., et al., *Characterization of Tunisian Mycobacterium tuberculosis rifampin-resistant clinical isolates.* J Clin Microbiol, 2007. **45**(9): p. 3095-7.

Insufficient data

98. Syre, H., et al., *Rapid colorimetric method for testing susceptibility of Mycobacterium tuberculosis to isoniazid and rifampin in liquid cultures.* J Clin Microbiol, 2003. **41**(11): p. 5173-7.

Case Control study design

99. Syre, H., et al., *Evaluation of the nitrate-based colorimetric method for testing the susceptibility of Mycobacterium tuberculosis to streptomycin and ethambutol in liquid cultures.* J Antimicrob Chemother, 2006. **57**(5): p. 987-91.

No testing for rifampicin resistance

100. Tho, D.Q., et al., *Comparison of MAS-PCR and GenoType MTBDR assay for the detection of rifampicin-resistant Mycobacterium tuberculosis.* Int J Tuberc Lung Dis, 2008. **12**(11): p. 1306-12.

Case Control study design

101. Tortoli, E. and F. Marcelli, *Use of the INNO LiPA Rif.TB for detection of Mycobacterium tuberculosis DNA directly in clinical specimens and for simultaneous determination of rifampin susceptibility.* Eur J Clin Microbiol Infect Dis, 2007. **26**(1): p. 51-5.

Insufficient data

102. Tovar, M., et al., *Improved diagnosis of pleural tuberculosis using the microscopic- observation drug-susceptibility technique.* Clin Infect Dis, 2008. **46**(6): p. 909-12.

No testing for rifampicin resistance

103. Traore, H., et al., *Direct detection of Mycobacterium tuberculosis complex DNA and rifampin resistance in clinical specimens from tuberculosis patients by line probe assay.* J Clin Microbiol, 2006. **44**(12): p. 4384-8.

Insufficient data

104. Van Deun, A., et al., *Drug susceptibility testing proficiency in the network of supranational tuberculosis reference laboratories.* Int J Tuberc Lung Dis, 2011. **15**(1): p. 116-24.

Case Control study design

105. Vijdea, R., et al., *Multidrug-resistant tuberculosis: rapid detection of resistance to rifampin and high or low levels of isoniazid in clinical specimens and isolates.* Eur J Clin Microbiol Infect Dis, 2008. **27**(11): p. 1079-86.

Case Control study design

106. Yajko, D.M., et al., *Colorimetric method for determining MICs of antimicrobial agents for Mycobacterium tuberculosis.* J Clin Microbiol, 1995. **33**(9): p. 2324-7.

Case Control study design

107. Zhang, L., et al., *Application of genotype MTBDRplus in rapid detection of the Mycobacterium tuberculosis complex as well as its resistance to isoniazid and rifampin in a high volume laboratory in Southern China.* Mol Biol Rep, 2011. **38**(3): p. 2185-92.

Lack of an accepted reference standard
